# The Functional Assessment of Chronic Illness Therapy–Fatigue (FACIT-Fatigue) scale in patients with axial spondyloarthritis: psychometric properties and clinically meaningful thresholds for interpretation

**DOI:** 10.1186/s41687-024-00769-x

**Published:** 2024-08-12

**Authors:** David Cella, Christine de la Loge, Fatoumata Fofana, Shien Guo, Alicia Ellis, Carmen Fleurinck, Ute Massow, Maxime Dougados, Victoria Navarro-Compán, Jessica A. Walsh

**Affiliations:** 1 Department of Medical Social Sciences, Feinberg School of Medicine, Northwestern University, Evanston, IL USA; 2 PCOM Analytics, Avallon, France; 3 Evidera, Zonneoordlaan, Netherlands; 4 Evidera, Waltham, MA USA; 5 UCB Pharma, Raleigh, NC USA; 6 UCB Pharma, Brussels, Belgium; 7 UCB Pharma, Monheim, Germany; 8 Department of Rheumatology, Hôpital Cochin, University Paris Cité, Paris, France; 9 Department of Rheumatology, La Paz University Hospital, IdiPaz, Madrid, Spain; 10 Salt Lake City Veterans Affairs Health and University of Utah Health, Salt Lake City, UT USA

**Keywords:** FACIT-Fatigue, Axial spondyloarthritis, Ankylosing spondylitis, Non-radiographic axial spondyloarthritis, Psychometric properties, Fatigue, Meaningful within-patient change, Meaningful between-group difference, Severity bands

## Abstract

**Background:**

Fatigue is an important symptom for most patients with axial spondyloarthritis (axSpA). The FACIT-Fatigue is a 13-item patient-reported outcome (PRO) instrument that has been used in axSpA clinical trials to measure fatigue severity and impact on daily activities. However, the psychometric properties of the FACIT-Fatigue are not fully evaluated across the entire spectrum of axSpA including non-radiographic axSpA (nr-axSpA) and radiographic axSpA (r-axSpA). This study determined: (1) the psychometric properties of the FACIT-Fatigue in nr-axSpA, r-axSpA, and the broad axSpA population and (2) FACIT-Fatigue scores representing meaningful within-patient change (MWPC), meaningful between-group differences, and cross-sectional severity bands.

**Methods:**

Data from two Phase 3 trials in adults with nr-axSpA (BE MOBILE 1; *N* = 254) and r-axSpA (BE MOBILE 2; *N* = 332) were analyzed pooled and separately to assess the psychometric properties of the FACIT-Fatigue. MWPC and meaningful between-group difference estimates were derived using anchor-based and distribution-based methods. Cross-sectional fatigue severity bands were estimated using logistic regression analysis.

**Results:**

The FACIT-Fatigue presented good internal consistency, adequate convergent and known-groups validity, and was sensitive to change over time across the full axSpA spectrum. A 5–11-point increase in FACIT-Fatigue score was estimated to represent a MWPC, with an 8-point increase selected as the responder definition. A 2.14–5.34-point difference in FACIT-Fatigue score change over a 16-week period was estimated to represent a small-to-medium meaningful between-group difference. FACIT-Fatigue score severity bands were defined as: none or minimal (>40), mild (>30 to ≤40), moderate (>21 to ≤30), and severe (≤21).

**Conclusions:**

These findings support the use of the FACIT-Fatigue as a fit-for-purpose measure to assess fatigue-related treatment benefit in axSpA clinical trials. The proposed score estimates and thresholds can guide FACIT-Fatigue score interpretation across the full axSpA spectrum.

**Trial registration:**

ClinicalTrials.Gov, NCT03928704. Registered 26 April 2019—Retrospectively registered, https://classic.clinicaltrials.gov/ct2/show/NCT03928704. ClinicalTrials.Gov, NCT03928743. Registered 26 April 2019—Retrospectively registered, https://classic.clinicaltrials.gov/ct2/show/NCT03928743.

**Supplementary Information:**

The online version contains supplementary material available at 10.1186/s41687-024-00769-x.

## Abstract (keymessage)

Fatigue is common in patients with axial spondyloarthritis (axSpA)—a painful, chronic disease that mainly affects the joints of the spine. Measuring fatigue in clinical trials is important to see if new treatments for axSpA help reduce this symptom. This study investigated whether a patient-completed measure of fatigue, the ‘FACIT-Fatigue’, is suitable for use by patients with either of the two main types of axSpA—radiographic axSpA (r-axSpA) and non-radiographic axSpA (nr-axSpA). This study was needed because we did not know if the FACIT-Fatigue is valid and reliable in both axSpA subtypes and because we are lacking information to interpret the FACIT-Fatigue score in axSpA. Psychometric analysis of clinical trial data showed that the FACIT-Fatigue accurately measures fatigue severity in both types of axSpA. The study additionally pinpointed what change in FACIT-Fatigue score can be used to define whether a treatment has reduced fatigue in a patient, or what constitutes a meaningful between-group difference. It also identified severity score bands that can be used to classify patients into those with no, mild, moderate, or severe fatigue. These findings support using the FACIT-Fatigue in axSpA clinical trials to measure how well treatments reduce fatigue.

**Supplementary Information:**

The online version contains supplementary material available at 10.1186/s41687-024-00769-x.

## Background

Axial spondyloarthritis (axSpA) is a chronic rheumatic disease involving inflammation of the sacroiliac joints and spine [1, 2]. Patients with axSpA can suffer from severe chronic back pain, stiffness, and fatigue, with symptoms typically emerging before 40 years of age [3–5]. AxSpA can be divided into two subtypes: non-radiographic axSpA (nr-axSpA) and radiographic axSpA (r-axSpA, also known as ankylosing spondylitis) [4]. Patients with nr-axSpA do not show definitive radiographic structural damage to the sacroiliac joints on pelvic radiographs, while patients with r-axSpA do. The degree of disability related to axSpA symptoms is, however, similar in nr-axSpA and r-axSpA [4–8].

In 2021, the Assessment of SpondyloArthritis international Society-Outcomes Measures in Rheumatology (ASAS-OMERACT) working group updated the core outcome set for r-axSpA to cover both r-axSpA and nr-axSpA (i.e., the full spectrum of axSpA disease) and address other advances in the field [9]. The working group selected seven core domains for axSpA and defined a core set of instruments to assess these domains [9, 10]. Fatigue, an important symptom for most axSpA patients [11–13], was included as a mandatory domain for all axSpA trials [9].

Multiple dimensions of fatigue can affect wellbeing in patients with axSpA [12, 13]. Multiple-item instruments such as the Functional Assessment of Chronic Illness Therapy Fatigue subscale (FACIT-Fatigue subscale, hereafter ‘FACIT-Fatigue’) [14–16] may therefore be useful to capture how patients with axSpA experience different aspects of fatigue. The FACIT-Fatigue is a 13-item patient-reported outcome (PRO) instrument used in many chronic conditions to measure the severity of fatigue and its impact on daily activities [14–17]. This instrument asks patients to rate statements about their feelings of fatigue (e.g., “I feel tired”) and vitality (e.g., “I have energy”), and the impact of their fatigue (e.g., “I need to sleep during the day”) over the past seven days on a 5-point Likert scale (0 [“not at all”] to 4 [“very much”]), and takes < 5 min to complete [18]. The 11 items that assess negatively phrased outcomes (e.g., I feel tired) are reverse-scored (i.e., a rating of 4 is converted to 0) and then all 13 item scores are summed to create a total score (range: 0–52 points) summarizing the patient’s level of fatigue. Higher total scores represent lower levels of fatigue.

The psychometric properties (i.e., reliability, construct validity, and responsiveness) of the FACIT-Fatigue have been evaluated in r-axSpA but not in nr-axSpA or the broad axSpA population [15, 19]. Understanding the psychometric properties of the FACIT-Fatigue in the broad axSpA population can help to determine whether the FACIT-Fatigue is a reliable, valid, and responsive measure for evaluating the severity of fatigue and its impact on daily activities across the axSpA disease spectrum.

Additionally, although meaningful within-patient change (MWPC) estimates for the FACIT-Fatigue have been proposed in r-axSpA [15], it is unclear whether such estimates could be also used in nr-axSpA or the broad axSpA population. Moreover, prior MWPC estimates were based on a single anchor without a clear understanding of what level of change on the anchor could be considered meaningful [15]. Further, there is no clear guidance on how to interpret between-group differences in FACIT-Fatigue score change, and no severity bands to distinguish different levels of fatigue cross-sectionally. Establishing such thresholds can help guide interpretation of the FACIT-Fatigue score in axSpA and may facilitate the use of this instrument to measure multiple aspects of fatigue in clinical trials or clinical practice.

This study aimed to: (1) assess the psychometric properties of the FACIT-Fatigue in nr-axSpA, r-axSpA, and the broad axSpA population and (2) estimate FACIT-Fatigue scores representing MWPC, meaningful between-group differences, and cross-sectional severity bands.

## Methods

### BE MOBILE 1 and BE MOBILE 2

Data from two Phase 3 randomized clinical trials (RCTs) in adults with active nr-axSpA (BE MOBILE 1; NCT03928704) and active r-axSpA (BE MOBILE 2; NCT03928743) [20–22] were used to assess the psychometric properties of the FACIT-Fatigue and estimate FACIT-Fatigue score thresholds for MWPC, meaningful between-group differences, and cross-sectional fatigue severity bands. BE MOBILE 1 and BE MOBILE 2 RCTs evaluated the efficacy and safety of bimekizumab (UCB4940), a monoclonal antibody that selectively inhibits interleukin-17F in addition to interleukin-17A, versus placebo [20–22].

Detailed eligibility criteria, methods, and results of these trials have been published [22]. Briefly, eligibility for BE MOBILE 1 required active, adult-onset nr-axSpA meeting ASAS classification criteria [22, 23], the absence of radiographic (i.e., x-ray) sacroiliitis, and inflammation defined by sacroiliitis on MRI and/or elevated C-reactive protein. Eligibility for BE MOBILE 2 required active r-axSpA and documented radiographic sacroiliitis fulfilling the 1984 modified NY criteria for r-axSpA [22, 24]. All patients in BE MOBILE 2 also fulfilled ASAS criteria. In both RCTs, patients completed a Baseline visit (Week 0) and were randomized to receive subcutaneous injections of bimekizumab 160 mg or placebo every four weeks during the 16-week double-blind treatment period [22].

In both RCTs, participants completed the FACIT-Fatigue and several other PRO instruments: Ankylosing Spondylitis Quality of Life (ASQoL); Bath Ankylosing Spondylitis Disease Activity Index (BASDAI); Bath Ankylosing Spondylitis Functional Index (BASFI); EQ-5D-3L; Medical Outcomes Study Sleep Scale—Revised (MOS Sleep-R); Patient’s Global Assessment of Disease Activity (PGADA); Patient Health Questionnaire-9 (PHQ-9); Short Form 36-Item Health Survey (SF-36); Total and Nocturnal Spinal Pain numeric rating scales; and Work Productivity and Activity Impairment Questionnaire—Specific Health Problem (WPAI:SHP) (Supplementary Methods Table 1).

Several clinical composite measures were derived in the RCTs, including the Assessment of SpondyloArthritis international Society (ASAS) clinical response criteria and the Ankylosing Spondylitis Disease Activity Score (ASDAS). ASAS response criteria assess improvement in axSpA based on improvement across four domains: patient global assessment by the PGADA score; pain by the Total Spinal Pain score; physical function by the BASFI score; and morning stiffness by the mean of BASDAI questions 5 and 6 [25]. ASAS 20% improvement (ASAS20) is defined as improvement of ≥20% and an absolute reduction of ≥1 unit on a 0–10 NRS in at least three of the four domains. In addition, ASAS20 requires no worsening in the remaining domain. ASAS 40% improvement (ASAS40) is defined as a relative improvement of ≥40% and an absolute reduction of ≥2 units on a 0–10 NRS in at least three of the four domains and no worsening in the remaining domain. ASAS partial remission (ASAS-PR) is defined as a score of ≤2 units on all four components. We categorized patients into mutually exclusive groups based on the most stringent ASAS response criteria that they met at Week 16: none of the ASAS response criteria; ASAS20 (but not ASAS40); ASAS40 (but not ASAS PR); or ASAS-PR (and ASAS40).

The ASDAS is a composite measure of axSpA disease activity comprised of an objective assessment of inflammation based on levels of C-reactive protein and several PRO measures: back pain by the BASDAI Q2, peripheral pain/swelling by the BASDAI Q3, morning stiffness by the BASDAI Q6, and global assessment by the PGADA score [26, 27]. This analysis categorized patients into mutually exclusive groups in two ways. Patients were categorized based on their change in ASDAS disease state (inactive disease [ID]: ASDAS < 1.3; low disease activity [LD]: ASDAS ≥ 1.3 to < 2.1; high disease activity [HD]: ASDAS ≥ 2.1 to ≤ 3.5; or very high disease activity [vHD]: ASDAS > 3.5) from Baseline to Week 16. Additionally, patients were categorized based on the most stringent ASDAS improvement criteria they met at Week 16: none, ASDAS-Clinically Important Improvement (ASDAS-CII; improvement ≥ 1.1), or ASDAS-Major Improvement (ASDAS-MI; improvement ≥ 2.0).

### Analysis overview

This study analyzed blinded data from the BE MOBILE 1 and BE MOBILE 2 studies, both separately and pooled across studies. Data were analyzed from Baseline and the double-blind treatment period; the FACIT-Fatigue was administered at Baseline, Week 4, and Week 16. Data were included from all randomized participants who had ≥ 1 non-missing FACIT-Fatigue score during the double-blind treatment period and were pooled across treatment and placebo groups (Supplementary Methods Table 1). Psychometric analyses were conducted to determine the internal consistency reliability, construct validity, and responsiveness of the FACIT-Fatigue. In addition, thresholds were estimated to aid interpretation of the FACIT-Fatigue: (1) MWPC estimates and the corresponding responder threshold were derived to interpret within-patient change over time, (2) meaningful between-group difference estimates were calculated to interpret between-group differences in change over time, and (3) fatigue severity bands were defined to help interpret levels of fatigue at a single point in time (i.e., cross-sectionally). Statistical analyses were performed using SAS^®^ software (Version 9.4).

### Psychometric analysis

#### Descriptive statistics of the FACIT-Fatigue

Frequency distributions of FACIT-Fatigue item scores and descriptive statistics of overall FACIT-Fatigue scores were calculated at Baseline and Week 16. Descriptive statistics included the proportion of patients with the lowest possible overall score (i.e., 0, referred to as floor) and the highest possible overall score (i.e., 52, referred to as ceiling).

#### Internal consistency reliability

Internal consistency reliability of the FACIT-Fatigue was assessed at Baseline and Week 16 using Cronbach’s alpha; coefficients ≥ 0.70 indicated acceptable internal consistency [28, 29] and ≥ 0.90 indicated excellent internal consistency [30, 31].

#### Construct validity

Construct validity of the FACIT-Fatigue was assessed via convergent validity and known-groups validity at Baseline and Week 16. Convergent validity assessed whether FACIT-Fatigue scores were sufficiently related to scores from 11 selected external outcome measures (e.g., BASDAI Q1-Fatigue). The 11 measurement sets were selected because they were hypothesized by the authors to be associated with fatigue, based on the content of the scale and the authors’ best judgment. The selected measures and the strengths and directions of their hypothesized correlations with the FACIT-Fatigue are listed in Supplementary Methods Table 2. Spearman’s correlation coefficients < 0.30 indicated weak correlation, ≥ 0.30 to < 0.70 indicated moderate correlation, ≥ 0.70 to < 0.90 indicated strong correlation, and ≥ 0.90 indicated very strong correlation [32].

Known-groups validity assessed how well the FACIT-Fatigue score could distinguish between groups of patients categorized into different severity or response categories on other related outcome measures [33]. Known groups were established based on the following outcome measures at Baseline and Week 16: ASQoL Item 7 (‘I am tired all the time’); ASQoL Item 12 (‘I get tired easily’); SF-36 Item 9g (‘Did you feel worn out during the past 4 weeks?’); SF-36 Item 9i (‘Did you feel tired during the past 4 weeks?’); PHQ-9 Item 4 (‘Over the last 2 weeks, how often have you been bothered by feeling tired or having little energy?’); and ASDAS disease states. These measures were selected because they were hypothesized by the authors to be useful in distinguishing groups of patients with different levels of fatigue. Selected criterion measures covered the same concept as fatigue or were expected to be associated with fatigue (e.g., ASDAS disease states), and used an ordinal or verbal rating scale that made scores easy to categorize into severity levels.

Mean FACIT-Fatigue scores were compared per known group, and the effect size (ES) of the difference in means between adjacent groups was estimated as the difference in means divided by the overall standard deviation (SD) at Baseline.

#### Responsiveness

We assessed the sensitivity of the FACIT-Fatigue to detect changes in fatigue over time (i.e., responsiveness) in two ways: using correlations between change scores from the FACIT-Fatigue and external criterion measures, and using analysis of covariance (ANCOVA) models. The external criterion measures were PHQ-9 Item 4, SF-36 Item 9g, SF-36 Item 9i, ASAS response levels, ASDAS disease states, and ASDAS improvement criteria (Supplementary Methods Table 3). These criterion measures were selected because they were hypothesized to be associated with fatigue or changes in fatigue. For each of the selected criterion measures, patients were categorized by level of change on that measure (e.g., up to 3 levels of improvement, no change, or worsening) from Baseline to Week 16. Correlations between change scores were calculated as Spearman’s correlations between changes from Baseline to Week 16 in the FACIT-Fatigue and changes from Baseline to Week 16 in the given criterion measure. Spearman’s correlations ≥ 0.30 indicated adequate responsiveness [34]. These correlations were examined to retain the external criterion measures to be used in the ANCOVA models for responsiveness and used to determine thresholds (or a range of thresholds) for MWPC and meaningful between-group differences. ANCOVA was used to compare the least squares (LS) mean changes from Baseline to Week 16 in FACIT-Fatigue score between levels of change for each retained criterion measure, adjusting for Baseline score. Known groups with a sample size < 15 were collapsed into an adjacent group.

### Estimating meaningful improvement and defining FACIT-Fatigue responders

#### Meaningful within-patient change estimates and responder definition threshold

MWPC estimates for the FACIT-Fatigue score were derived using anchor-based methods as the primary approach; distribution-based methods provided supportive information [35–37]. In the anchor-based methods, summary statistics of FACIT-Fatigue score change from Baseline to Week 16 were calculated for each level of change within each anchor. Anchors were selected considering the strength of their correlation with changes in the FACIT-Fatigue as determined in the responsiveness analyses, their content, their use as established clinical endpoints, and their ease of interpretation. In the distribution-based methods, the standard error of measurement (SEM) and 0.5 × SD and 0.8 × SD of the FACIT-Fatigue scores at Baseline were calculated. Additionally, empirical cumulative distribution function (eCDF) curves were generated based on FACIT-Fatigue score change from Baseline to Week 16. Curves were generated separately for each level of change within each retained anchor to determine which level of improvement on the given anchor could be considered meaningful. eCDF curves were examined by visual inspection to identify anchor levels of change to be used to derive MWPC estimates. In cases where the eCDF curve for the first level of improvement on the anchor was clearly separated from the eCDF curve of the no change group, the distribution statistics for this first level of improvement were used. In cases where the first level of improvement was not clearly separated from the no change group, the next level of improvement was considered.

Estimates from the anchor-based and distribution-based approaches were then triangulated to determine a range of values that might constitute a MWPC. A threshold to define FACIT-Fatigue responders (i.e., a responder definition [RD]) was selected from the range of MWPC estimates.

#### Meaningful between-group difference estimates

Meaningful between-group difference estimates were calculated to guide comparison of changes over time in FACIT-Fatigue scores between two groups (e.g., treatment vs placebo groups in an RCT). As there is no consensus on how to derive a meaningful between-group difference, meaningful between-group difference estimates were determined using anchor-based and distribution-based methods [38–40]. ANCOVA was used to estimate the difference in LS mean change from Baseline in FACIT-Fatigue score at Week 16 between ‘no change’ and one-level of improvement on each selected anchor, with Baseline FACIT-Fatigue score used as the covariate. Following the same approach as in the MWPC analysis, anchors were selected considering the strength of their correlation with changes in the FACIT-Fatigue as determined in the responsiveness analyses, their content, their use as established clinical endpoints, and their ease of interpretation. Distribution-based estimates (SEM, 0.2 × SD, 0.5 × SD, and 0.8 × SD) provided supportive information.

### Fatigue severity bands

FACIT-Fatigue score cutoffs defining different levels of fatigue severity (i.e., fatigue severity bands) cross-sectionally were estimated using logistic regression based on the following criterion measures at pooled timepoints: SF-36 Item 9g, SF-36 Item 9i, and ASDAS disease states (Supplementary Methods Table 1). These criterion measures were selected because they were hypothesized to reflect different levels of fatigue severity and were easy to interpret. Receiver operating characteristic (ROC) curves were plotted to evaluate the classification performance of each regression model and to help select optimal score cutoff values.

Logistic regression analyses were based on data pooled from all timepoints where the FACIT-Fatigue and the given criterion measure were assessed at the same study visit. Analyses using SF-36 Item 9g and SF-36 Item 9i as criterion measures were conducted using Baseline and Week 16 data combined, and analyses using ASDAS disease states as criterion measures were conducted using Baseline, Week 4, and Week 16 data combined.

Each criterion measure was dichotomized by collapsing adjacent ordinal categories to define “responders” vs. “non-responders”. We then ran logistic regression, using the dichotomized criterion measure as the response (i.e., dependent variable) and the continuous FACIT-Fatigue score as the predictor (i.e., independent variable). Three separate logistic regression models were run for each criterion measure to assess each possible dichotomization based on adjacent categories: 1 vs. 2–4, 1–2 vs. 3–4, and 1–3 vs. 4.

The area under the curve (AUC) was estimated for each ROC curve; an AUC ≥ 0.70 indicated satisfactory accuracy [41]. The optimal FACIT-Fatigue cutoff score from each ROC analysis was determined based on the highest value of the Youden index (sensitivity + specificity– 1) [42]. Identified cutoff score ranges were examined by a team of clinicians, psychometricians, and statisticians to determine if they were suitable to guide interpretation of fatigue severity. If the ranges were too narrow or would lead to inconsistent results with the proposed RD, alternative cutoff values were considered by taking into account the Youden index value, the percent of patients classified correctly, and ensuring that patients improving by 2 levels of severity would be classified as FACIT-Fatigue responders. The final proposed values were endorsed by the team.

## Results

### Patients’ characteristics

All randomized participants in BE MOBILE 1 and BE MOBILE 2 had ≥ 1 non-missing FACIT-Fatigue score during the double-blind treatment period and were therefore included in this analysis. The pooled sample included 586 patients with axSpA: 254 patients with nr-axSpA from BE MOBILE 1 and 332 patients with r-axSpA from BE MOBILE 2 (Table 1). Patients in the pooled data set had a mean age of 39.5 (SD, 11.9) years. Most patients were male (65%), White (83%), and living in Europe (80%).

On average, patients had experienced their first axSpA symptoms 11.5 (SD, 9.9) years ago and had been diagnosed with axSpA for 5.2 (SD, 7.2) years. The nr-axSpA and r-axSpA RCTs differed in gender distribution (54 vs 72% male), disease duration ≥ 2 years (39 vs 60% of patients), and time since diagnosis (3.6 [SD, 5.8] years vs. 6.4 [SD, 7.9] years). Almost all (99%) patients had high or very high ASDAS disease activity at Baseline.

**Table 1:** Patients’ demographics and disease characteristics for the pooled and separate studies

	Pooled	BE MOBILE 1 (nr-axSpA)	BE MOBILE 2 (r-axSpA)
n at Baseline	586	254	332
Age in years, Mean (SD)	39.5 (11.9)	38.9 (11.5)	40.0 (12.3)
Female, %	35.5	45.7	27.7
Region, %			
North America	4.6	7.1	2.7
Eastern Europe	52.4	56.7	49.1
Western Europe	27.8	25.2	29.8
Asia	15.2	11.0	18.4
Race, %			
Black	0.7	1.2	0.3
White	82.9	86.2	80.4
Asian	14.5	11.0	17.2
Missing	1.9	1.6	2.1
BMI group, %			
≤18.5 kg/m^2^	2.0	1.6	2.4
≥18.5 to <25 kg/m^2^	38.9	38.6	39.2
≥25 to <30 kg/m^2^	31.9	31.9	31.9
≥30 kg/m^2^	27.1	28.0	26.5
HLA-B27 positive, %	82.1	77.6	85.5
Time since first axSpA symptoms in years			
Mean (SD)	11.5 (9.9)	9.0 (8.8)	13.5 (10.3)
Median	8.8	5.5	10.8
Q1, Q3	3.9, 17.1	2.3, 12.8	5.9, 19.0
Time since axSpA diagnosis in years			
Mean (SD)	5.2 (7.2)	3.6 (5.8)	6.4 (7.9)
Median	2.1	1.3	3.6
Q1, Q3	0.53, 6.84	0.45, 4.03	0.66, 8.77
Duration of disease since diagnosis, %			
<2 years	48.8	60.6	39.8
≥2 years	51.2	39.4	60.2
Baseline ASDAS disease state, %			
Inactive disease (<1.3)	0.0	0.0	0.0
Low disease activity (≥1.3 to <2.1)	1.2	1.6	0.9
High disease activity (≥2.1 to ≤3.5)	39.8	40.2	39.5
Very high disease activity (>3.5)	59.0	58.3	59.6

*Abbreviations ASDAS* Ankylosing Spondylitis Disease Activity Score, *BMI* body mass index, *HLA-B27* human leukocyte antigen B27, *nr-axSpA* non-radiographic axial spondyloarthritis, *Q* quartile, *r-axSpA* radiographic axial spondyloarthritis, *SD* standard deviation

### Psychometric properties

#### Descriptive statistics of the FACIT-Fatigue

**Fig. 1:**
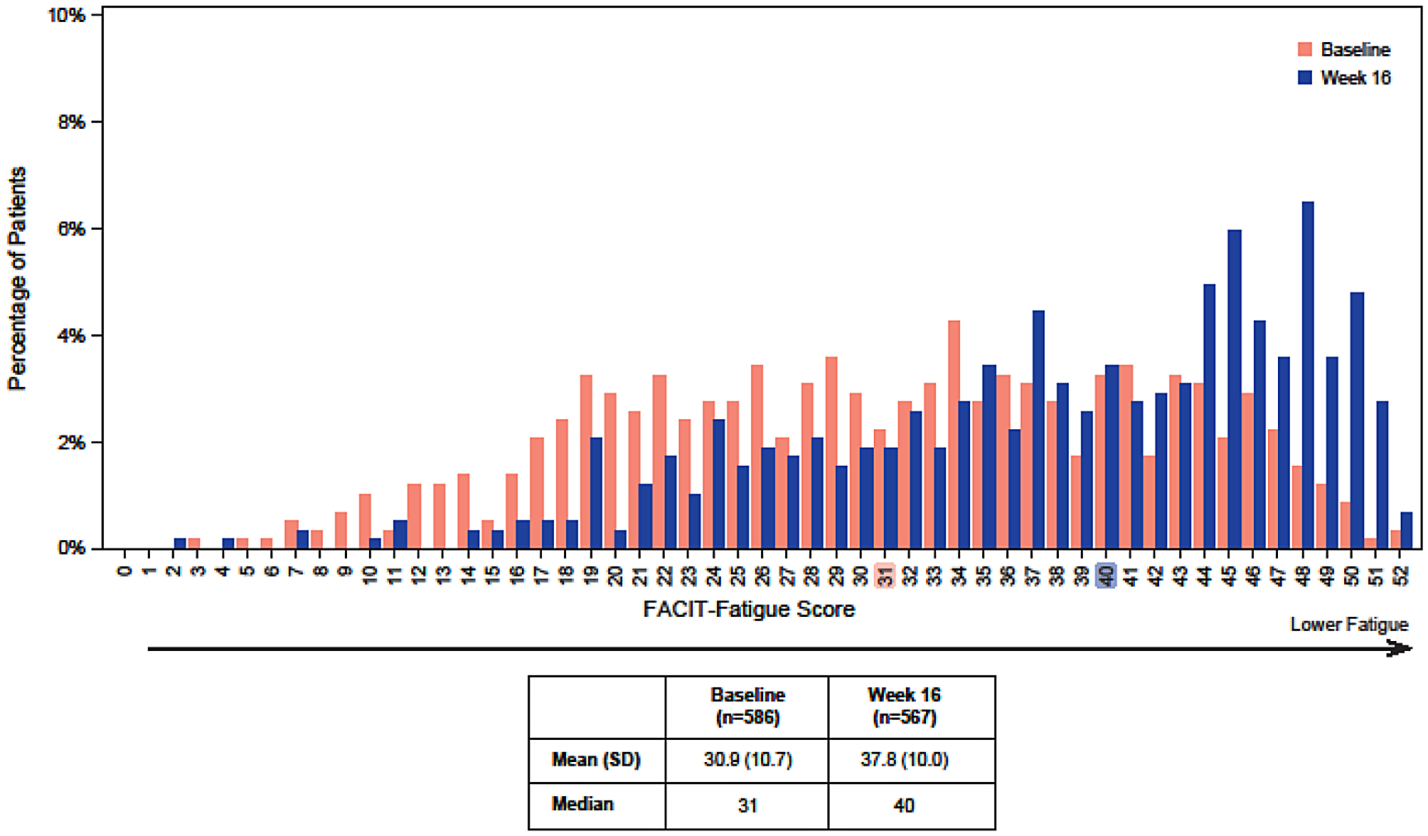
FACIT-Fatigue score distributions at Baseline and Week 16 in the pooled studies. Distribution of FACIT-Fatigue scores at Baseline (orange) and Week 16 (blue) in the pooled studies. Higher scores indicate lower levels of fatigue. Mean (SD) and median scores are listed by assessment visit. *Abbreviations FACIT-Fatigue* Functional Assessment of Chronic Illness Therapy—Fatigue, *SD *standard deviation

At Baseline and Week 16, patients endorsed the full range of responses for each FACIT-Fatigue item. The distribution of FACIT-Fatigue scores covered the full spectrum of the score range (Fig. 1). No problematic floor or ceiling effects were observed at either timepoint; at Baseline, 0.0% of patients were at floor and 0.34% were at ceiling, and at Week 16, 0.0% were at floor and 0.71% at ceiling. This suggests that the scale could capture improvement and worsening. The frequency distributions of FACIT-Fatigue item scores and descriptive statistics of overall FACIT-Fatigue scores were mostly similar between the nr-axSpA and r-axSpA studies at Baseline (Supplementary Results Fig. 1; Supplementary Results Table 1). FACIT-Fatigue descriptive statistics varied between the two studies at Week 16 (Supplementary Results Fig. 1), such that higher scores (i.e., lower levels of fatigue) were observed in the r-axSpA study. These differences at Week 16 may reflect the greater proportion of patients randomized to bimekizumab vs. placebo in the r-axSpA study (2:1) than in the nr-axSpA study (1:1). Because of the similar item-level descriptive results across the two studies at Baseline, results of the psychometric analyses are reported from the pooled studies. Psychometric results from the separate studies are provided in the Supplementary Results.

#### Internal consistency reliability

Cronbach’s alphas were 0.94 at both Baseline and Week 16, suggesting excellent internal consistency reliability of the FACIT-Fatigue score. The separate nr-axSpA and r-axSpA studies had nearly identical Cronbach’s alphas (Supplementary Results Table 2).

#### Convergent validity

At Baseline and Week 16, FACIT-Fatigue scores were significantly correlated with each of the selected external outcome measures using Spearman’s rank correlations (all *p* < 0.001) (Table 2). All correlations were in the expected direction and most aligned with the *a priori* hypotheses, supporting the convergent validity of the FACIT-Fatigue.

**Table 2:** Convergent validity in pooled studies: Correlations between FACIT-Fatigue scores and selected outcome measures

Outcome measure	Baseline		Week 16	
	Correlation between FACIT-Fatigue score and selected outcome measure		Correlation between FACIT-Fatigue score and selected outcome measure	
	n	r	n	r
BASDAI Q1-Fatigue	586	−0.48	567	−0.66
SF-36 Vitality	586	0.79	567	0.84
PHQ-9 Item 4 score	586	−0.29	567	−0.45
Total Spinal Pain score	586	−0.36	567	−0.53
Nocturnal Spinal Pain score	586	−0.38	567	−0.52
MOS Sleep-R domain scores				
Sleep Disturbance	586	0.54	567	0.53
Sleep Problems Index II	586	0.7	567	0.72
BASDAI total score	586	−0.50	567	−0.62
BASFI score	586	−0.58	567	−0.58
PGADA score	586	−0.42	567	−0.51
ASDAS	586	−0.36	567	−0.51
ASQoL total score	586	−0.83	567	−0.80
Other SF-36 domains				
Physical Functioning	586	0.65	567	0.64
Role Physical	586	0.63	567	0.72
Bodily Pain	586	0.6	567	0.64
General Health	586	0.49	567	0.55
Social Functioning	586	0.62	567	0.66
Role Emotional	586	0.39	567	0.49
Mental Health	586	0.56	567	0.65
Physical Component Summary	586	0.61	567	0.67
Mental Component Summary	586	0.54	567	0.61
EQ-5D VAS score	586	0.48	567	0.56
WPAI:SHP domain scores				
Absenteeism	431	−0.34	425	−0.35
Presenteeism	393	−0.55	384	−0.61
Overall productivities loss	393	−0.55	384	−0.62
Percent of activities impairment	586	−0.59	567	−0.63
PHQ-9 total score	586	−0.27	567	−0.44

Spearman’s rank correlations. All correlations were statistically significant at *p *< 0.001

Correlation coefficients with absolute values < 0.30 were considered as weak, ≥ 0.30 and < 0.70 as moderate, ≥ 0.70 and < 0.90 as strong, and ≥ 0.90 as very strong

*Abbreviations ASDAS* Ankylosing Spondylitis Disease Activity Score, *ASQoL* ankylosing spondylitis quality of life, *BASDAI* Bath Ankylosing Spondylitis Disease Activity Index, *BASDAI Q1-Fatigue* BASDAI single-item question on fatigue, *BASFI* bath ankylosing spondylitis functional index, *EQ-5D VAS* EQ-5D visual analogue scale, *FACIT-Fatigue* Functional Assessment of Chronic Illness Therapy—Fatigue, *MOS Sleep-R* medical outcomes study sleep scale—revised, *PGADA* patient’s global assessment of disease activity, *PHQ-9* patient health questionnaire—9 items, *SF-36* short form 36-item health survey, *WPAI:SHP* work productivity and activity impairment questionnaire-specific health problem—specific health problem

FACIT-Fatigue scores showed strong (|*r*| ≥ 0.70 and < 0.90) correlations at both Baseline and Week 16 with the ASQoL total score, SF-36 Vitality score, and MOS Sleep-R Sleep Problems Index II (Table 2). FACIT-Fatigue scores were also strongly correlated with the SF-36 Role Physical score at Week 16. Moderate (|*r*| ≥ 0.30 and < 0.70) correlations were observed between FACIT-Fatigue scores and most other considered outcomes at both Baseline and Week 16, including the BASDAI Q1-Fatigue (Baseline, −0.48; Week 16, −0.66). Similar results were observed for the separate studies (Supplementary Results Table 3).

#### Known-groups validity

**Fig. 2:**
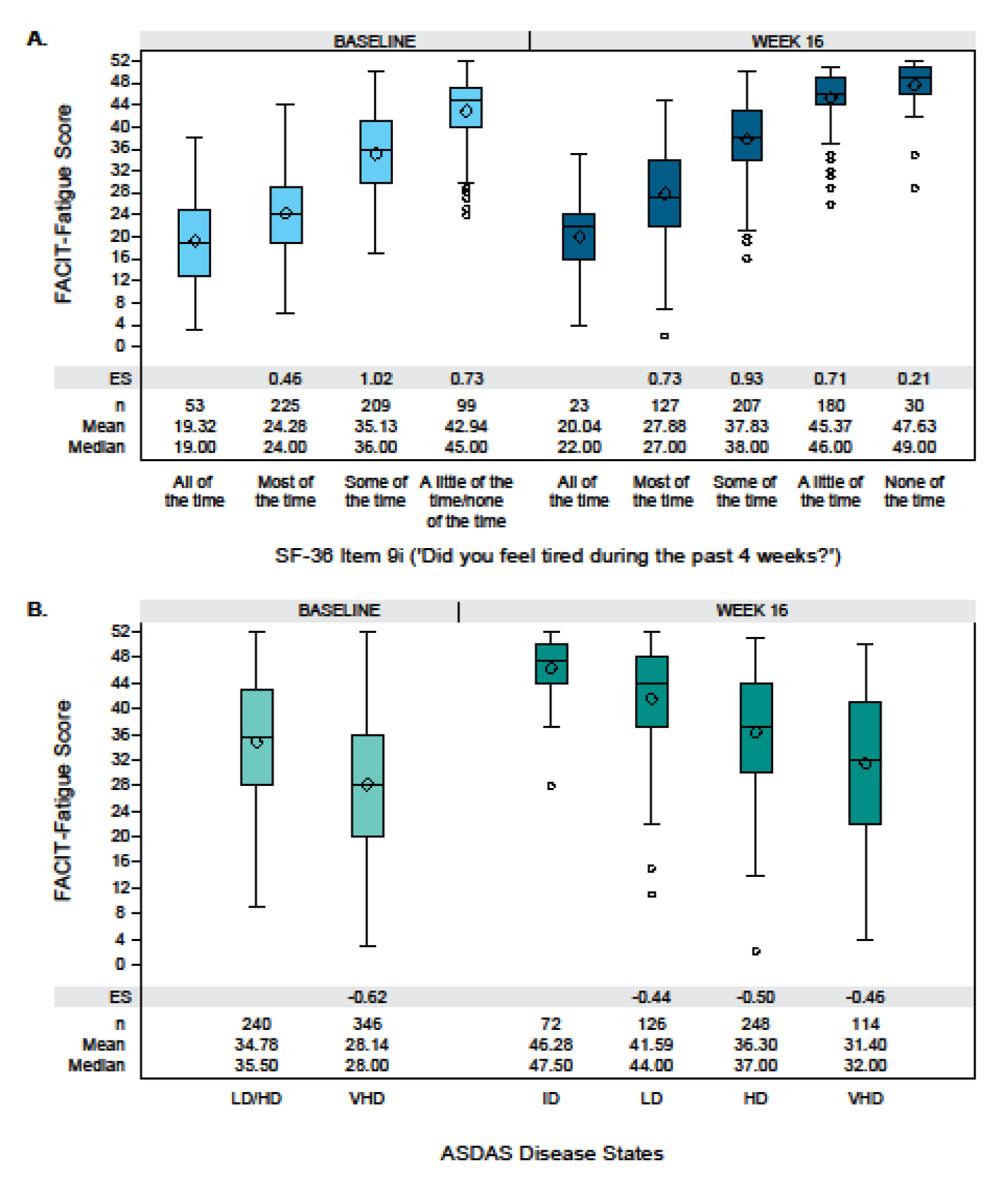
Known-groups validity in pooled studies: Distributions of FACIT-Fatigue scores by external parameter. Boxplots show differences in FACIT-Fatigue score distributions between adjacent known groups at Baseline and Week 16 for (**A**) SF-36 Item 9i and (**B**) ASDAS disease states for the pooled studies. Lines in boxplots represent median scores, diamonds represent mean scores, and whiskers indicate 95% CIs. Known groups with a sample size < 15 were collapsed into the adjacent group. *Abbreviations ASDAS* Ankylosing Spondylitis Disease Activity Score, *CI* confidence interval, *ES* effect size, *FACIT-Fatigue* Functional Assessment of Chronic Illness Therapy—Fatigue, *HD* ASDAS high disease activity, *ID* ASDAS inactive disease, *LD* ASDAS low disease activity, *SF-36* Short form 36-item health survey, *VHD* ASDAS very high disease activity

At both Baseline and Week 16, the FACIT-Fatigue score discriminated between groups of patients known to be clinically different based on ASQoL Item 7, ASQoL Item 12, SF-36 Item 9 g, SF-36 Item 9i, PHQ-9 Item 4, or ASDAS disease states (Fig. 2; Supplementary Results Table 4). All differences in FACIT-Fatigue means between adjacent known groups were statistically significant (all p < 0.001 with ES > 0.2) and in the expected direction. Similar results were observed for the individual studies (Supplementary Results Table 5).

#### Responsiveness

FACIT-Fatigue score change from Baseline to Week 16 was significantly correlated with the change captured with each external criterion measure over the same time period (all *p *< 0.001). Correlations were moderate for five of the six considered criterion measures: SF-36 Item 9 g (*r*: − 0.56), SF-36 Item 9i (*r*: − 0.55), ASAS response levels (*r*: − 0.41), ASDAS disease states (*r*: − 0.36), ASDAS-CII/ASDAS-MI (*r*: − 0.40). The correlation was weaker for PHQ-9 Item 4 (*r*: − 0.27). Three of the criterion measures were retained for further analyses: change in SF-36 Item 9i, ASAS response levels, and change in ASDAS disease states. Criterion measures were retained based on their strength of correlation in addition to their content, use as established clinical endpoints, and ease of interpretation. Further explanation is provided in the Supplementary Results.

**Fig. 3:**
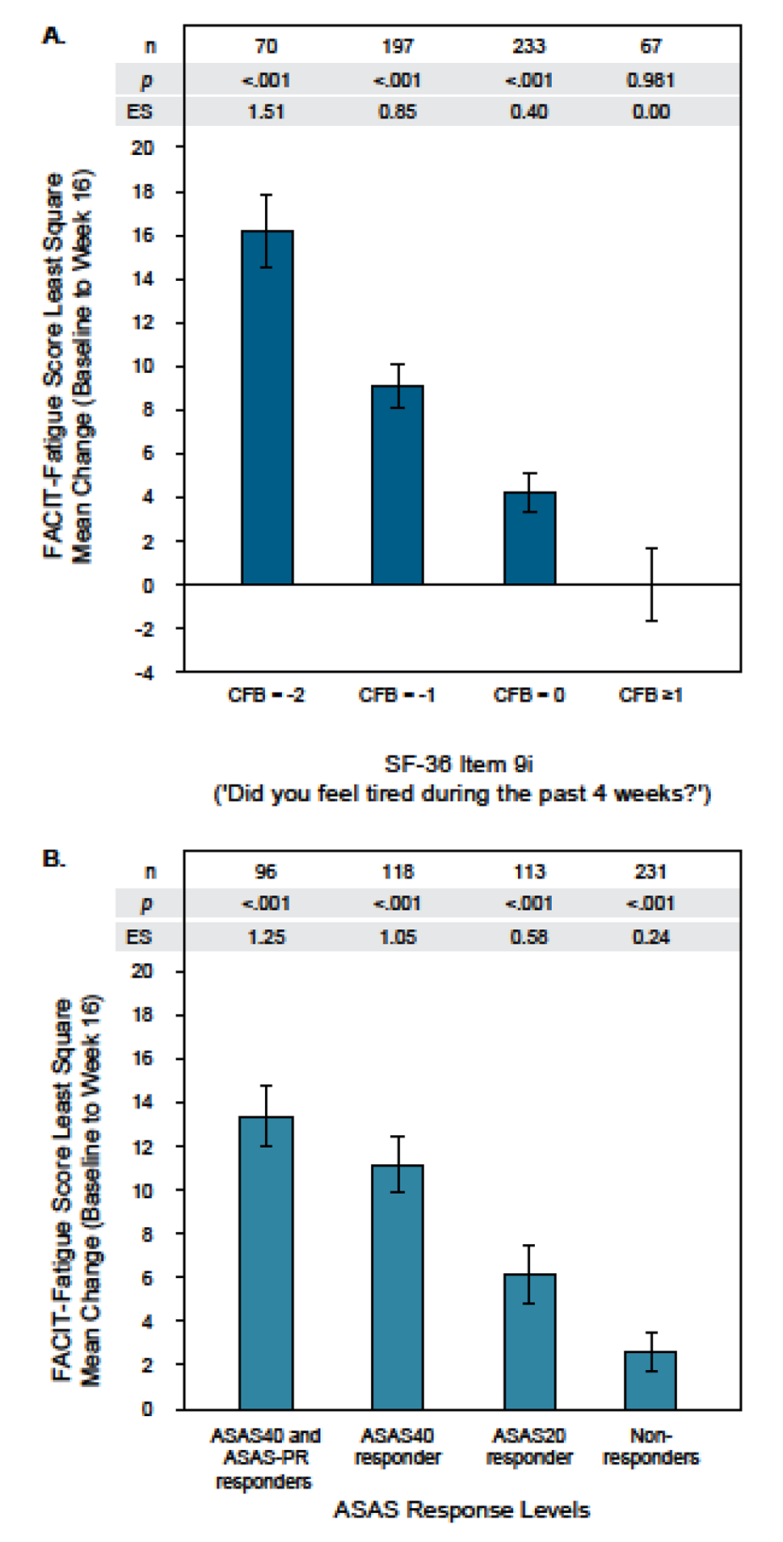
Responsiveness in pooled studies: FACIT-Fatigue score least square mean change by criterion measure level of change. Bar graphs show LS mean change (95% CI) on the FACIT-Fatigue for each level of change based on (**A**) SF-36 Item 9i and (**B**) ASAS response level criterion measures. ASAS20 is defined as improvement of ≥ 20% and an absolute improvement of ≥ 1 unit on a 0–10 numeric rating scale in ≥ 3 of the four ASAS domains. In addition, ASAS20 requires no worsening in the remaining domain (relative worsening of ≥ 20% and absolute worsening of ≥ 1 unit). ASAS40 is defined as a relative improvement of ≥ 40% and an absolute improvement of ≥ 2 units on a 0–10 NRS in ≥ 3 of the four ASAS domains and no worsening at all in the remaining domain. ASAS-PR is defined as a score of ≤ 2 units on a 0–10 NRS in all four domains. *Abbreviations ASAS* assessment of spondyloarthritis international society, *CFB* change from baseline, *CI* confidence interval, *ES* effect size, *FACIT-Fatigue* Functional Assessment of Chronic Illness Therapy—Fatigue, *ES* effect size, *LS* least squares, *SF-36* short form 36-item health survey

LS mean score changes from Baseline to Week 16 were significantly different across all levels of change for the retained criterion measures (overall F-test: *p *< 0.001), supporting the ability of the FACIT-Fatigue score to detect changes over time (Fig. 3; Supplementary Results Table 6). Similar results were observed for the individual studies (Supplementary Results Table 7A–7B).

### Estimates for interpreting change in the FACIT-Fatigue score

#### MWPC thresholds

**Fig. 4:**
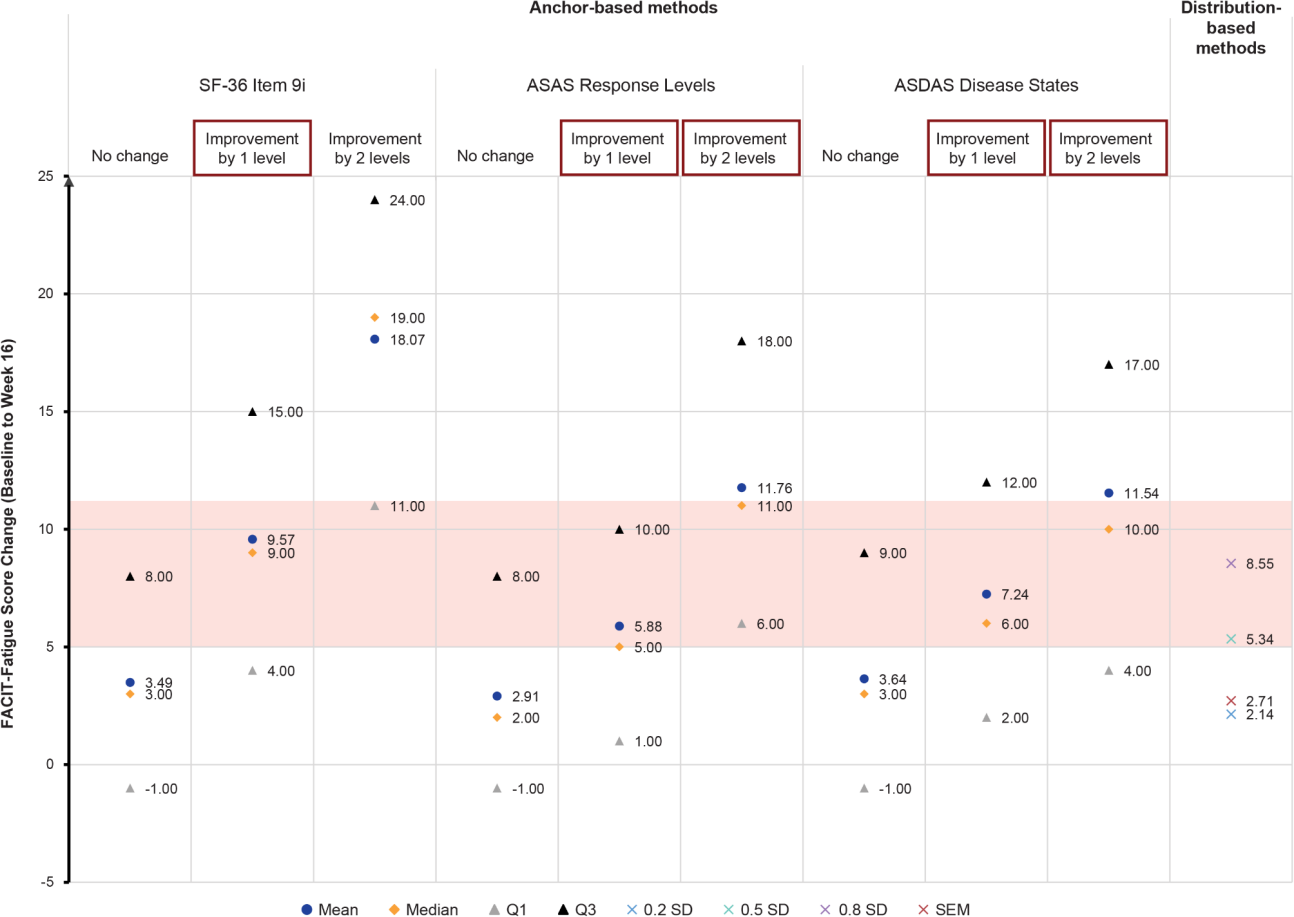
Anchor-based and distribution-based estimates of meaningful within-patient change threshold for FACIT-Fatigue score change. Estimates of MWPC using anchor- and distribution-based approaches based on data from the pooled studies. The highlighted range from 5–11 points represents a MWPC based on triangulating from selected estimates. *Abbreviations ASAS* assessment of spondyloarthritis international society, *ASDAS* Ankylosing Spondylitis Disease Activity Score, *FACIT-Fatigue* Functional Assessment of Chronic Illness Therapy—Fatigue, *MWPC* meaningful within-patient change, *Q* quartile, *SD* standard deviation, *SEM* standard error, *SF-36* short form 36-item health survey

Several anchor levels of change were retained: one-level improvement on SF-36 Item 9i; ASAS response levels for both one-level and two-level improvement; and both one-level and two-level improvement in ASDAS disease states (Supplementary Results Figures 2–4; Supplementary Results Table 8). These anchor levels of change were retained primarily based on their eCDF curves; detailed rationale for retaining these levels is provided in the Supplementary Results. A 5-point to 11-point increase in FACIT-Fatigue score from Baseline to Week 16 was estimated to represent a MWPC based on the median change in the retained anchor levels of change (Fig. 4; Supplementary Results Table 8). The mid-point of this MWPC range—an 8-point increase in the FACIT-Fatigue score—is proposed as a useful primary value for the RD. The distribution-based approach showed that one SEM of the FACIT-Fatigue score at Baseline was 2.71, 0.50 × SD of the FACIT-Fatigue score at Baseline was 5.34, and 0.80 × SD was 8.55. Therefore, an 8-point increase can be considered to represent a somewhat large change and thus a conservative estimate for defining response.

#### Meaningful between-group differences estimates

The estimated difference in LS mean changes from Baseline to Week 16 in the FACIT-Fatigue was 4.81 (95% confidence interval [CI]: 3.48, 6.13; ES: 0.45) based on the SF-36 Item 9i, 3.53 (95% CI: 1.95, 5.12; ES: 0.33) based on ASAS response levels, and 3.65 (95% CI: 2.27, 5.02; ES: 0.34) based on ASDAS disease states. The distribution-based analysis estimated that small-to-medium-size effects would correspond to score differences between 2.14 (0.20 × SD), indicating small-size effects, and 5.34 (0.50 × SD), indicating medium-size effects. Based on the LS mean change differences and distribution-based estimates, a between-group difference of 2.14–5.34 points on the FACIT-Fatigue score was estimated to represent a small-to-medium meaningful between-group difference.

### Fatigue severity bands

**Fig. 5:**
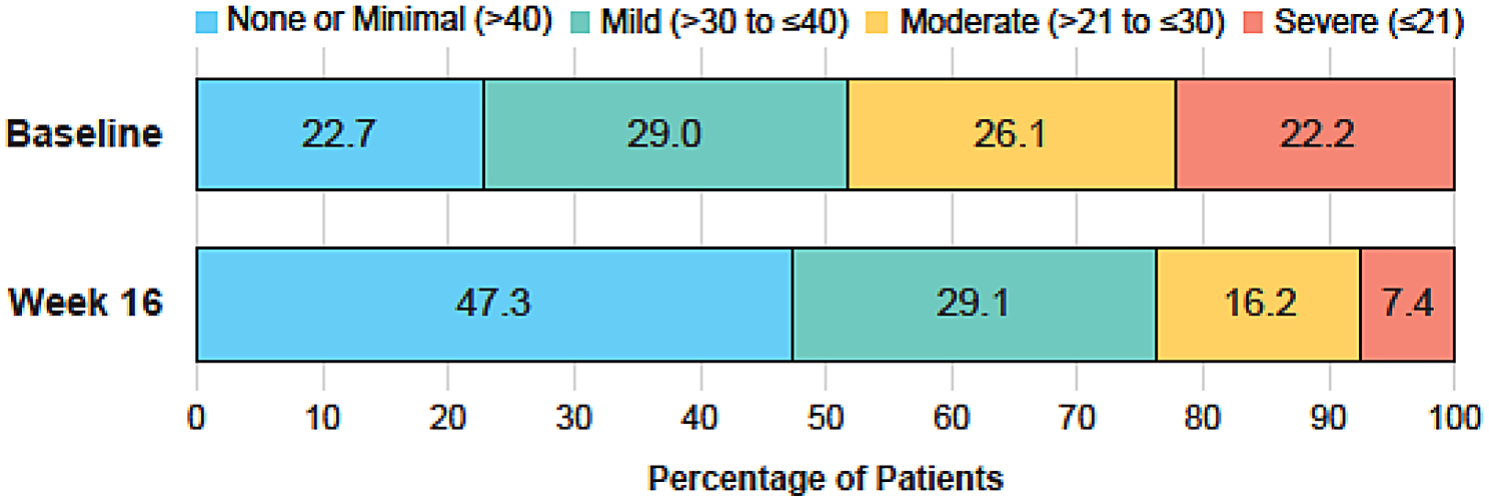
FACIT-Fatigue score severity bands: Percentage of patients falling within each fatigue severity band. Proportion of patients falling within each fatigue severity band at Baseline and Week 16, using the final FACIT-Fatigue severity band cutoffs. *Abbreviation FACIT-Fatigue* Functional Assessment of Chronic Illness Therapy—Fatigue

The AUC statistics were > 0.70 for all the logistic regression analyses, indicating good discriminant power of the FACIT-Fatigue score to classify different levels of severity defined by the selected anchors (Supplementary Results Table 9). Based on the maximum Youden indices from the selected anchors, the optimal cutoff scores to define different severity bands for the FACIT-Fatigue score were initially defined as: none or minimal (>40), mild (>30 to ≤ 40), moderate (>27 to ≤ 30), and severe (≤27). However, the score range initially identified for the moderate level of fatigue (>27 to ≤ 30) was narrow and could lead to inconsistent results with the proposed RD of eight points. For this reason, a revised score range of > 21 to ≤ 30 was proposed for moderate fatigue. This range was proposed because, for the anchor with the highest AUC (SF-36 Item 9g), the cut-offs presenting the highest Youden index were 22 (Youden Index, 0.64) and 25 (0.65), but the 22 cut-off presented a higher percentage of subjects correctly classified (86.9 vs 80.9%). At Baseline, roughly equal proportions of patients fell within each of the proposed (revised) severity bands; at Week 16, a greater proportion of patients fell within bands representing lower levels of fatigue (Fig. 5).

## Discussion

Quantitative analyses of two RCTs in nr-axSpA and r-axSpA showed that the FACIT-Fatigue had good internal consistency, adequate convergent and known-groups validity, and was sensitive to change over time. Additionally, a 5–11 point increase in the FACIT-Fatigue score was estimated to represent a MWPC. This MWPC range was derived based on anchor-based and distribution-based analyses, as recommended by the US Food and Drug Administration guidance on PRO and clinical outcome assessment measures [36]. An 8-point score increase (i.e., the midpoint of the MWPC range) is proposed as the RD. This corresponds to 15.1% of the scale range, which exceeds the requirement from the Institute for Quality and Efficiency in Health Care that RDs comprise at least 15% of the scale range [43]. Furthermore, a between-group difference of 2.14–5.34 points on the FACIT-Fatigue score was estimated to represent a small-to-medium meaningful difference between patient groups. Severity bands for the FACIT-Fatigue score were proposed to classify patients according to their fatigue over the past seven days: none or minimal (>40), mild (>30 to ≤ 40), moderate (>21 to ≤ 30), and severe (≤21).

This study confirmed that the FACIT-Fatigue has good psychometric properties in nr-axSpA, r-axSpA, and the broad axSpA population, which extends prior findings of the scale’s psychometric performance in r-axSpA [15, 19]. The MWPC range (5–11 points) and associated RD (8 points) proposed in this study are higher (i.e., requiring greater change) than thresholds identified in the literature for r-axSpA and other rheumatic diseases like psoriatic arthritis and rheumatoid arthritis, which range from 3–6.3 points [15, 19, 44, 45]. The more stringent values proposed here are likely due to differences in study design and analytical methods. Cella et al. (2022) analyzed RCT data in r-axSpA using all follow-up assessment timepoints (Week 2—Week 12 in Study 1 and Week 2—Week 16 in Study 2) [15] whereas the present study used Baseline and Week 16 timepoints. The longer timeframe in the present study may have allowed for greater treatment-related changes in fatigue to occur. Further, thresholds in Cella et al. (2022) were based on a single PGADA NRS anchor, whereas this study considered a set of PRO and clinical anchors for which levels of meaningful change could be clearly understood. The thresholds proposed in Cella et al. (2005) for rheumatoid arthritis (3–4 points) were derived from distribution-based estimates using ESs of 0.2–0.5 SD units, whereas the distribution-based estimates used in this study were based on ESs above 0.5 SD units [45]. The thresholds proposed in Revicki et al. (2011) for r-axSpA (3–4 points) were not empirically-based but likely guided by the ranges reported in rheumatoid arthritis (3–4 points) [19, 45].

The meaningful between-group difference estimates obtained in this study (2.14–5.34 points) are consistent with the between-groups clinically important difference scores suggested for r-axSpA by Revicki et al. (2011) (4–5 points) [19] and those estimated in patients with psoriatic arthritis (2.25–3.29 points) [44] and confirmed in patients with rheumatoid arthritis (3–4 points) [45].

To our knowledge, this is the first study to propose FACIT-Fatigue severity bands in axSpA. Such cross-sectional severity bands may help clinicians distinguish different levels of fatigue among their patients and provide appropriate care. The proposed cutoff score of 40 to distinguish patients with axSpA who have ‘none or minimal’ fatigue from those who have ‘mild’ fatigue aligns with the mean FACIT-Fatigue subscale score of 43.6 in the US general population [46] and the cutoff score of 43 previously proposed to distinguish the US general population from patients with anemia and cancer [46]. The proposed cutoff score of 30 to distinguish ‘moderate’ from ‘mild’ fatigue is also similar to the previously defined cutoff of ≤ 30 indicating clinically significant fatigue in cancer patients [47]. In addition, the proposed cutoff score of 21 to define ‘severe’ fatigue in axSpA is similar to typical scores seen in patients with anemia and cancer (mean: 23.9; median: 23.0) [46].

This study was also the first to validate the FACIT-Fatigue in nr-axSpA as well as the first in the broad axSpA population. This study was based on data from nearly 600 patients with axSpA and included roughly even proportions of patients with nr-axSpA (43.3%) and r-axSpA (56.7%). Demographic and disease characteristics in the sample were representative of nr-axSpA and r-axSpA disease in RCTs [4]. Overall mean, distribution, and range of FACIT-Fatigue scores were generally similar between nr-axSpA and r-axSpA studies at Baseline, supporting the pooled analyses, but differed somewhat between the two studies at Week 16—likely due to the difference in randomization schemes between the two studies (i.e., patients were randomized 2:1 to active treatment in the r-axSpA study and 1:1 in the nr-axSpA study). The r-axSpA study therefore had more patients receiving active treatment and hence a higher mean increase in FACIT-Fatigue score (i.e., reduced fatigue).

This study focused on the FACIT-Fatigue because this scale has several strengths for assessing fatigue in axSpA. First, it is a well-established measure of fatigue that has been psychometrically evaluated and used extensively across chronic diseases, including rheumatic conditions. Second, because it is a multi-item measure assessing various aspects of the fatigue experience (e.g., physical fatigue, mental fatigue, fatigability), it is well-suited to assess the complex, multi-faceted patient experience of fatigue [13, 48].

Although initially considered, the FACIT-Fatigue was not fully evaluated by the ASAS-OMERACT working group as a potential core instrument to measure fatigue because of limited available information [10]. The ASAS-OMERACT working group instead evaluated and selected the BASDAI Q1-Fatigue as the recommended instrument for assessing fatigue in axSpA [9, 10]. The BASDAI Q1-Fatigue is a single item measure where participants rate their “overall level of fatigue/tiredness” over the last week using a 0–10 NRS. The BASDAI Q1-Fatigue has shown good psychometric properties and has been well-implemented in r-axSpA RCTs [10]. A comparison of the BASDAI Q1-Fatigue and the FACIT-Fatigue in the broad axSpA population was beyond the scope of this study but could be an interesting direction for future work.

This study had several limitations. First, test-retest reliability was not assessed due to expected changes in fatigue over the four-week and 12-week intervals between assessments [13, 49]. However, the FACIT-Fatigue has demonstrated acceptable test-retest reliability over a two-week interval in ‘stable’ patients with r-axSpA [15] and in patients with psoriatic arthritis [44]. In addition, content validity was not assessed and has not been confirmed in axSpA, although it has been confirmed in other rheumatic diseases [44, 50]. Third, the feasibility of the FACIT-Fatigue was not assessed. Nonetheless, there was a > 96% completion rate of the FACIT-Fatigue in both BE MOBILE studies, suggesting that patients had no difficultly completing the questionnaire. Another limitation is that the logistic regression models used to generate the ROC curves assumed the independence of repeated measures within patients. The impact of this assumption on the cut-offs was not fully explored in the analysis. Finally, we ran several separate binary logistic regressions based on different dichotomizations of each criterion measure, but other methods exist that may have allowed us to estimate the accuracy of these ordinal criterion measures more efficiently [51, 52].

## Conclusions

Analysis of two RCTs in nr-axSpA and r-axSpA demonstrated that the FACIT-Fatigue has good psychometric properties across the full spectrum of axSpA. Further, score estimates and thresholds were proposed to guide interpretation of FACIT-Fatigue scores in axSpA. Overall, these findings support the use of the FACIT-Fatigue as a fit-for-purpose measure to assess fatigue-related treatment benefit in axSpA RCTs.

### Electronic supplementary material

Below is the link to the electronic supplementary material.

Supplementary Material 1

## Data Availability

The datasets analyzed during the current study are available from the corresponding author on reasonable request.

## Abbreviations

ANCOVA: Analysis of covariance
ASAS: Assessment in SpondyloArthritis international Society
ASDAS: Ankylosing Spondylitis Disease Activity Score
ASQoL: Ankylosing Spondylitis Quality of Life
AUC: area under the curve
axSpA: axial spondyloarthritis
BASDAI: Bath Ankylosing Spondylitis Disease Activity Index
BASFAI: Bath Ankylosing Spondylitis Functional Index
eCDF: empirical cumulative distribution function
ES: effect size
EQ-5D-3L: European Quality of Life 5 Dimensions 3 Level Version
FACIT-Fatigue: Functional Assessment of Chronic Illness Therapy Fatigue subscale
LS: least squares
MWPC: meaningful within-patient change
MOS Sleep-R: Medical Outcomes Study Sleep Scale—Revised
nr-axSpA: non-radiographic axial spondyloarthritis
NRS: numeric rating scale
PHQ-9: Patient Health Questionnaire-9
PGADA: Patient’s Global Assessment of Disease Activity
PRO: patient-reported outcome
r-axSpA: radiographic axial spondyloarthritis
RCT: randomized controlled trial
RD: responder definition
SD: standard deviation
SEM: standard error of measurement
SF-36: Short Form 36-Item Health Survey
WPAI:SHP: Work Productivity and Activity Impairment Questionnaire—Specific Health Problem
