# Supplementary material for: The Functional Assessment of Chronic Illness Therapy–Fatigue (FACIT-Fatigue) scale in patients with axial spondyloarthritis: psychometric properties and clinically meaningful thresholds for interpretation
Source: J Patient Rep Outcomes. 2024 Aug 12;8:92. doi: 10.1186/s41687-024-00769-x (PMC11319684; doi:10.1186/s41687-024-00769-x)
Supplement: Supplementary file 1 — Supplementary Material 1 [file 41687_2024_769_MOESM1_ESM.docx]

**Supplementary Materials for: The Functional Assessment of Chronic Illness Therapy–Fatigue (FACIT-Fatigue) scale in patients with axial spondyloarthritis: Psychometric properties and clinically meaningful thresholds for interpretation**

# Supplementary Methods

## Overview of PRO assessments in BE MOBILE 1 and BE MOBILE 2

### **Supplementary Methods Table 1. Patient-reported outcome measures used to assess the psychometric properties of FACIT-Fatigue.**

|  | | | | **Characteristics** | | | |
| --- | --- | --- | --- | --- | --- | --- | --- |
| **PRO measure** | **Periods administered ^a^** | **Main concept covered** | **Items** | **Recall period** | **Scoring** | **Score range** | **Score interpretation** |
| **FACIT-Fatigue** | B; W4; W16 | Fatigue | 13 | Past 7 days | FACIT-Fatigue subscale score | 0–52 | Higher scores indicate lower levels of fatigue |
| **BASDAI** | B; W1; W2; W4; W8; W12; W16 |  |  |  |  |  |  |
| *BASDAI total score* |  | Disease activity | 6 | Last week | BASDAI total score | 0–10 | Lower scores indicate lower levels of disease activity |
| *BASDAI Q1-Fatigue* |  | Fatigue | 1 | Last week | BASDAI Q1-Fatigue | 0–10 | Lower scores indicate lower levels of fatigue |
| **BASFI** | B; W1; W2; W4; W8; W12; W16 | Physical function | 10 | Last week | BASFI score | 0–10 | Lower scores indicate better physical function |
| **ASQoL** | B; W4; W8; W12; W16 | HRQoL | 18 | At the moment | ASQoL total score | 0–18 | Lower scores indicate better HRQOL |
| **PGADA** | B; W1; W2; W4; W8; W12; W16 | Disease activity | 1 | Last week | PGADA score | 0–10 | Lower scores indicate lower levels of disease activity |
| **Total and Nocturnal Spinal Pain Scale** | B; W1; W2; W4; W8; W12; W16 | Pain intensity | 2 | NR for total spinal pain  ‘At night’ for nocturnal spinal pain | Total spinal pain score;  Nocturnal spinal pain score | 0–10 | Lower scores indicate lower levels of pain |
| **SF-36** | B; W8; W16 | HRQoL | 36 | Past 4 weeks | PF, RP, BP, GH, VT, SF, RE, MH, HT, PCS, MCS | 0–100 | Higher scores indicate better health state |
| **EQ-5D-3L** | B; W4; W16 | Health state | 6 | At day of completion | Item scores: Mobility, self-care, usual activities, pain/discomfort, anxiety/depression  EQ-VAS | Item scores: 0–2;  EQ–VAS: 0–100 | Item scores: lower scores indicate lower problems  EQ-VAS: higher scores indicate better health state |
| **PHQ-9** | B; W4; W8; W12; W16 | Depression severity | 10 | Last 2 weeks | PHQ-9 total score | 0–27 | Lower scores indicate less severe depression |
| **WPAI:SHP** | B; W16 | Impact of axSpA on work productivity and daily activities | 6 | Past 7 days | Percent of work time missed;  Percent of impairment while working;  Percent of overall work impairment; Percent of activity impairment | 0–100 | Lower percents indicate lower activity impairment |
| **MOS Sleep-R** | B; W8; W16 | Sleep | 12 | Past 4 weeks | Sleep Disturbance, Sleep Adequacy, Sleep Quantity, Sleep Somnolence, Snoring, Shortness of Breath, or Headache | Sleep Quantity: no range;  Others: 0–100 | Higher scores indicate higher level of the measured attribute |

Abbreviations: ASQoL, Ankylosing Spondylitis Quality of Life; B, Baseline visit; BASDAI, Bath Ankylosing Spondylitis Disease Activity Index; BASFI, Bath Ankylosing Spondylitis Functional Index; BP, Bodily Pain; EQ-VAS, EQ-Visual Analog Scale; FACIT-Fatigue, Functional Assessment of Chronic Illness Therapy - Fatigue; GH, General Health; HRQoL, health related quality of life; HT, health transition; MCS, Mental Component Summary; MH, Mental Health; MOS Sleep-R, Medical Outcomes Study Sleep Scale - Revised; NR, Not reported; PCS, Physical Component Summary; PF, Physical Functioning; PGADA, Patient’s Global Assessment of Disease Activity; PHQ-9, Patient Health Questionnaire-9; PRO, patient-reported outcome; RE, Role Emotional; RP, Role Physical; SF, Social Functioning; SF-36, Short Form 36-Item Health Survey; VAS, visual analogue scale; VT, Vitality; W, week; WPAI:SHP, Work Productivity and Activity Impairment Questionnaire – Specific Health Problem.

^a^ Time points when the instruments were administered at Baseline and during the double-blind period (up to Week 16) in BE MOBILE 1 and BE MOBILE 2 RCTs. B indicates Baseline visit; W indicates study week.

## Hypothesized convergent validity

### **Supplementary Methods Table 2. Convergent validity for the pooled studies: Hypothesized relationships between FACIT-Fatigue and other PRO measurements.**

| **Measurement set** | **Strength of correlation** | **Direction of correlation** |
| --- | --- | --- |
| BASDAI Q1-Fatigue | moderate-to-strong | negative |
| SF-36 VT score | moderate-to-strong | positive |
| PHQ-9 Item 4 score | moderate-to-strong | negative |
| Total Spinal Pain and Nocturnal Spinal Pain score | moderate | negative |
| MOS Sleep-R domain scores (Sleep Disturbance and Sleep Problems Index II) | weak-moderate | negative |
| BASDAI total score | moderate | negative |
| BASFI score | moderate | negative |
| PGADA score | weak-to-moderate | negative |
| ASDAS-CRP | weak-moderate | negative |
| ASQoL total score | moderate | negative |
| Other SF-36 domains ^a^ | relatively weaker | positive |
| EQ-5D VAS score | moderate | positive |
| WPAI:SHP domain scores |  |  |
| Absenteeism | relatively weaker | negative |
| Presenteeism | moderate | negative |
| Overall productivities loss | moderate | negative |
| Percent of activities impairment | moderate | negative |

Abbreviations: ASDAS-CRP, Ankylosing Spondylitis Disease Activity Score – C-reactive protein; ASQoL, Ankylosing Spondylitis Quality of Life; BASDAI, Bath Ankylosing Spondylitis Disease Activity Index; BASFI, Bath Ankylosing Spondylitis Functional Index; EQ-5D VAS, EQ-Visual Analog Scale; FACIT-Fatigue, Functional Assessment of Chronic Illness Therapy–Fatigue; MOS Sleep-R, Medical Outcomes Study Sleep Scale - Revised; PCS, Physical Component Summary; PGADA, Patient’s Global Assessment of Disease Activity; PHQ-9, Patient Health Questionnaire-9; PRO, patient-reported outcome; SF-36, Short Form 36-Item Health Survey; VT, vitality; WPAI:SHP, Work Productivity and Activity Impairment Questionnaire – Specific Health Problem.

^a^ Other SF-36 domains: physical functioning, role physical, bodily pain, general health, social functioning, role emotional, mental health, health transition, physical component summary, and mental component summary.

## Responsiveness

## Supplementary Methods Table 3. Response categorizations by external criterion measure (change from Baseline to Week 16).

|  | Improvement by: | | | |  | No Improvement: | |
| --- | --- | --- | --- | --- | --- | --- | --- |
|  | **≥4 Levels (-4)** | **3 Levels (-3)** | **2 Levels (-2)** | **1 Level (-1)** |  | **No Change (0)** | **Worsening ^a^** |
| *PRO criterion measures* |  |  |  |  |  |  |  |
| PHQ-9 Item 4 ^b^ | n/a | -3 | -2 | -1 |  | 0 | ≥1 |
|  | | | | | | | |
| SF-36 items |  | | | | | | |
| *SF-36 Item 9g* ^b^ | 4 | 3 | 2 | 1 |  | 0 | ≤-1 |
| *SF-36 Item 9i* ^b^ | 4 | 3 | 2 | 1 |  | 0 | ≤-1 |
|  |  |  |  |  |  |  |  |
| *Clinical criterion measures* |  |  |  |  |  |  |  |
| ASAS Response Levels ^c^ | n/a | ASAS40 and ASAS-PR | ASAS40 only | ASAS20 only |  | Neither ASAS20, ASAS40, or ASAS-PR | |
|  |  |  |  |  |  |  |  |
| ASDAS criteria | | | | | | | |
| *ASDAS Disease States ^d^* | n/a | 3 | 2 | 1 |  | 0 | ≤- 1 |
| *ASDAS Improvement* ^e^ | n/a | n/a | ASDAS-MI | ASDAS-CI only |  | Neither ASDAS-CI or ASDAS-MI | |

Abbreviations: ASAS, Assessment of SpondyloArthritis international Society; ASDAS, Ankylosing Spondylitis Disease Activity Score; ASDAS-CII, Ankylosing Spondylitis Disease Activity Score - Clinically Important Improvement; ASDAS-MI, Ankylosing Spondylitis Disease Activity Score - Major Improvement; ASAS-PR, Assessment of SpondyloArthritis international Society - Partial Remission; HD, ASDAS high disease activity; ID, ASDAS inactive disease; LD, ASDAS low disease activity; PHQ-9, Patient Health Questionnaire-9; SF-36, Short Form 36-Item Health Survey; VHD, ASDAS very high disease activity.

^a^ Combined across levels of worsening.

^b^ Expressed as change in score from Baseline to Week 16.

^c^ Defined by meeting responder criteria for ASAS20, ASAS40, and/or ASAS-PR at Week 16. ASAS20 is defined as improvement of ≥20% and an absolute improvement of ≥1 unit on a 0–10 numeric rating scale in ≥3 of the four ASAS domains. In addition, ASAS20 requires no worsening in the remaining domain (relative worsening of ≥20% and absolute worsening of ≥1 unit). ASAS40 is defined as a relative improvement of ≥40% and an absolute improvement of ≥2 units on a 0–10 NRS in ≥3 of the four ASAS domains and no worsening at all in the remaining domain. ASAS-PR is defined as a score of ≤2 units on a 0–10 NRS in all four domains.

^d^ Expressed as levels of change in ASDAS disease activity states (ID, LD, HD, VHD) from Baseline to Week 16, with positive values indicating improvement (e.g., change from ID to VHD indicates improvement by 3 levels) and negative values indicating worsening.

^e^ According to ASDAS-CII/ ASDAS-MI criteria at Week 16.

# Supplementary Results

## Selection of external criterion measures to be retained for ANCOVA and MWPC threshold analyses

The SF-36 Item 9i was retained as an external criterion measure because it had a moderately strong correlation with FACIT-Fatigue changes from Baseline to Week 16, it is a PRO measuring fatigue (“tired”), and it comprises multiple levels of change that can be easily interpreted and understood. ASAS response levels was retained as a criterion measure because of its correlation with FACIT-Fatigue changes from Baseline to Week 16, because it is a common measure of short-term improvement in clinical trials, and because one of its levels (‘ASAS40’) was the primary endpoint in the BE MOBILE 1 and BE MOBILE 2 RCTs. ASDAS disease states was retained as a criterion measure because of its correlation with FACIT-Fatigue changes from Baseline to Week 16, because it is a well-established composite measure to assess disease severity in axSpA, and because its response categories comprise multiple levels of change that can be easily interpreted and understood.

## MWPC thresholds

Patients who improved by one level in SF-36 Item 9i (‘How much of the time during the past 4 weeks did you feel tired’) from Baseline to Week 16 had a median change of 9 points on the FACIT-Fatigue during this period (**Figure 4; Supplementary Results Table 8**). Patients who improved by two levels had a median change of 19 points. Using one or two levels of improvement on SF-36 9i as an anchor yielded large ESs (one level: 0.90; two levels: 1.69), indicating that either of these may be suitable anchors for determining MWPC. In addition, both the one-level and two-level improvement estimated cumulative density function (eCDF) curves showed a consistent and clear separation across SF-36 Item 9i change categories in the expected direction and were clearly separated from the no-change curve (**Supplementary Results Figure 2**). However, a two-level improvement on SF-36 9i would likely provide too stringent a threshold for clinical improvement using the FACIT-Fatigue, so the one-level improvement on SF-36 Item 9i was selected to help define the MWPC threshold.

Patients who improved by one ASAS response level (i.e., ASAS20 responders) had a median change of 5 points on the FACIT-Fatigue, whereas those who improved by two ASAS response levels (i.e., ASAS40 responders without ASAS-PR) had a median change of 11 points (**Supplementary Results Table 8**). Using one ASAS response level as an anchor yielded a moderate ES (0.55); using two response levels yielded a large ES (1.10). eCDF curves showed some overlaps between the one-level improvement curve and the no-change curve (**Supplementary Results Figure 3**), which suggests that estimating the MWPC threshold from the one-level improvement may not be large enough to define responders. On the other hand, the two-level improvement curve was clearly separated from the no-change and one-level improvement curve but would lead to an overly stringent criterion. Based on these considerations, the FACIT-Fatigue score for defining MWPC is proposed to fall between the 5-point median from one-level improvement and the 11-point median from two-level improvement.

Patients who improved by one level on ASDAS disease states (e.g., from VHD to HD or from HD to LD) had a median change of 6 points on the FACIT-Fatigue (**Supplementary Results Table 8**). Patients who improved by two levels had a median change of 10 points. Using one level of improvement on ASDAS diseases states as an anchor yielded a moderate ES (0.68) while using two levels of improvement as an anchor yielded a large ES (1.08). eCDF curves showed a consistent separation across ASDAS change categories in the expected direction (**Supplementary Results Figure 4**). Both the one- and two-level improvement curves were separated from the no-improvement curve. Given the proximity between the no-improvement curve and the one-level improvement curve, both one-level and two-level ASDAS improvements were considered when defining MWPC and RD thresholds.

## Supplementary Results Figures

### **Supplementary Results Figure 1. Separate studies FACIT-Fatigue score distributions at Baseline and Week 16.**

Distribution of FACIT-Fatigue scores at Baseline (orange) and Week 16 (blue) for the (**A**) nr-axSpA and (**B**) r-axSpA studies. Higher scores indicate lower levels of fatigue. Median scores for each study and timepoint are denoted by color-coded shading on the horizontal axes. Abbreviations: FACIT-Fatigue, Functional Assessment of Chronic Illness Therapy - Fatigue; nr-axSpA, non-radiographic axial spondyloarthritis; r-axSpA, radiographic axial spondyloarthritis; SD, standard deviation.

### **Supplementary Results Figure 2. eCDF plot of FACIT-Fatigue changes from Baseline to Week 16 by SF-36 Item 9i change category.**

Empirical cumulative distribution function plot describing patients’ changes in FACIT-Fatigue score from Baseline to Week 16 by their changes in SF‑36 Item 9i. SF-36 Item 9i asks patients to answer, “How much of the time during the past 4 weeks did you feel tired?”. Curves separate patients whose SF-36 9i score worsened (red), did not change (grey), improved by 1 level (blue), or improved by 2 levels (teal). Abbreviations: eCDF, empirical cumulative distribution function; FACIT-Fatigue, Functional Assessment of Chronic Illness Therapy - Fatigue; SF-36, Short Form 36-Item Health Survey.

### **Supplementary Results Figure 3. eCDF plot of FACIT-Fatigue changes from Baseline to Week 16 by ASAS response level.**

Empirical distribution function plot describing patients’ changes in FACIT-Fatigue score from Baseline to Week 16 by their ASAS response level. Curves separate patients who: did not meet any of the three ASAS response criteria (red); met ASAS20 response criteria but did not meet ASAS40 and ASAS-PR response criteria (blue); met ASAS40 response criteria but did not meet ASAS-PR response criteria (teal); or met both ASAS40 and ASAS-PR response criteria (green). Abbreviations: ASAS, Assessment of SpondyloArthritis international Society; ASAS-20, ASAS 20% response criteria; ASAS-40, ASAS 40% response criteria; ASAS-PR, ASAS partial remission; eCDF, empirical cumulative distribution function; FACIT-Fatigue; Functional Assessment of Chronic Illness Therapy – Fatigue.

### **Supplementary Results Figure 4. eCDF plot of FACIT-Fatigue changes from Baseline to Week 16 by ASDAS disease state change category.**

Empirical distribution function plot describing patients’ changes in FACIT-Fatigue score from Baseline to Week 16 by their change in ASDAS disease state. Patients were categorized into mutually exclusive groups corresponding to their change in ASDAS disease states. ASDAS score <1.3 indicated inactive disease, ≥1.3 to <2.1 indicated low disease activity, ≥2.1 to ≤3.5 indicated high disease activity, and >3.5 indicated very high disease activity. Abbreviations: ASDAS, Ankylosing Spondylitis Disease Activity Score; eCDF, empirical cumulative distribution function; FACIT-Fatigue, Functional Assessment of Chronic Illness Therapy ­– Fatigue.

## Supplementary Results Tables

### **Supplementary Results Table 1. FACIT-Fatigue descriptive statistics at Baseline and Week 16 from the pooled and separate studies.**

|  | **Pooled** | **BE MOBILE 1**  **(nr-axSpA)** | **BE MOBILE 2**  **(r-axSpA)** |
| --- | --- | --- | --- |
| **Baseline** |  |  |  |
| N | 586 | 254 | 332 |
| Mean (SD) | 30.85 (10.68) | 30.06 (10.68) | 31.46 (10.67) |
| Median | 31 | 30.50 | 32 |
| Q1, Q3 | 22, 40 | 22, 39 | 23, 40 |
| 10^th^, 90^th^ percentile | 17, 45 | 16, 44 | 17, 46 |
| Min, Max | 3, 52 | 3, 50 | 7, 52 |
| Observed range | 49 | 47 | 45 |
|  |  |  |  |
| **Week 16** |  |  |  |
| N | 567 | 246 | 321 |
| Mean (SD) | 37.79 (10.02) | 36.4 (10.44) | 38.85 (9.57) |
| Median | 40 | 37 | 41 |
| Q1, Q3 | 31, 46 | 30, 45 | 32, 47 |
| 10^th^, 90^th^ percentile | 23, 49 | 22, 49 | 24, 49 |
| Min, Max | 2, 52 | 2, 51 | 7, 52 |
| Observed range | 50 | 49 | 45 |

Abbreviations: FACIT-Fatigue, Functional Assessment of Chronic Illness Therapy–Fatigue; nr-axSpA, non-radiographic axial spondyloarthritis; Q, quartile; r-axSpA, radiographic axial spondyloarthritis; SD, standard deviation.

### **Supplementary Results Table 2. Internal consistency reliability of the FACIT-Fatigue by study.**

|  | **BE MOBILE 1 (nr-axSpA)** | **BE MOBILE 2 (r-axSpA)** |
| --- | --- | --- |
| **Baseline** |  |  |
| N | 254 | 332 |
| Cronbach’s alpha | 0.94 | 0.94 |
| **Week 16** |  |  |
| N | 246 | 321 |
| Cronbach’s alpha | 0.94 | 0.94 |

Abbreviations: FACIT-Fatigue, Functional Assessment of Chronic Illness Therapy - Fatigue; nr-axSpA, non-radiographic axial spondyloarthritis; r-axSpA, radiographic axial spondyloarthritis.

### **Supplementary Results Table 3. Convergent validity by study: Correlations between FACIT-Fatigue score and other selected outcome measures.**

| **Outcome measure** | **BE MOBILE 1 (nr-axSpA)** | | **BE MOBILE 2 (r-axSpA)** | |
| --- | --- | --- | --- | --- |
|  | **Baseline (n=254)** | **Week 16 (n=246)** | **Baseline (n=332)** | **Week 16 (n=321)** |
| BASDAI Q1-Fatigue | -0.51 | -0.69 | -0.45 | -0.63 |
| SF-36 Vitality | 0.76 | 0.83 | 0.81 | 0.84 |
| PHQ-9 Item 4 score | -0.35 | -0.47 | -0.24 | -0.43 |
| Total Spinal Pain score | -0.37 | -0.57 | -0.36 | -0.49 |
| Nocturnal Spinal Pain score | -0.37 | -0.53 | -0.38 | -0.50 |
| MOS Sleep-R domain scores |  |  |  |  |
| Sleep Disturbance | 0.53 | 0.51 | 0.54 | 0.53 |
| Sleep Problems Index II | 0.70 | 0.74 | 0.69 | 0.69 |
| BASDAI total score | -0.50 | -0.63 | -0.50 | -0.59 |
| BASFI score | -0.54 | -0.60 | -0.61 | -0.56 |
| PGADA score | -0.42 | -0.52 | -0.41 | -0.49 |
| ASDAS | -0.35 | -0.53 | -0.36 | -0.50 |
| ASQoL total score | -0.82 | -0.79 | -0.83 | -0.79 |
| Other SF-36 domains |  |  |  |  |
| Physical Functioning | 0.60 | 0.62 | 0.68 | 0.64 |
| Role Physical | 0.63 | 0.73 | 0.63 | 0.72 |
| Bodily Pain | 0.59 | 0.62 | 0.61 | 0.64 |
| General Health | 0.47 | 0.58 | 0.51 | 0.53 |
| Social Functioning | 0.59 | 0.61 | 0.65 | 0.69 |
| Role Emotional | 0.36 | 0.50 | 0.42 | 0.48 |
| Mental Health | 0.49 | 0.64 | 0.61 | 0.67 |
| Physical Component Summary | 0.60 | 0.65 | 0.61 | 0.67 |
| Mental Component Summary | 0.47 | 0.58 | 0.59 | 0.64 |
| EQ-5D VAS score | 0.48 | 0.61 | 0.48 | 0.52 |
| WPAI:SHP domain scores |  |  |  |  |
| Absenteeism^a^ | -0.41 | -0.28 | -0.27 | -0.39 |
| Presenteeism^a^ | -0.58 | -0.58 | -0.53 | -0.63 |
| Overall productivities loss^a^ | -0.61 | -0.58 | -0.51 | -0.64 |
| Percent of activities impairment | -0.58 | -0.60 | -0.60 | -0.64 |
| PHQ-9 total score | -0.33 | -0.46 | -0.22 | -0.43 |

All correlations were statistically significant at p<0.001

Correlations in absolute values <0.30 were considered as weak, between 0.30 and 0.70 as moderate, 0.70 to 0.90 as strong, and ≥0.90 as very strong.

Abbreviations: ASDAS, Ankylosing Spondylitis Disease Activity Score; ASQoL, Ankylosing Spondylitis Quality of Life; BASDAI, Bath Ankylosing Spondylitis Disease Activity Index; BASFI, Bath Ankylosing Spondylitis Functional Index; EQ-5D VAS, EQ-5D Visual Analogue Scale; FACIT-Fatigue, Functional Assessment of Chronic Illness Therapy – Fatigue; MOS Sleep-R, Medical Outcomes Study Sleep Scale - Revised; nr-axSpA, non-radiographic axial spondyloarthritis; PGADA, Patient’s Global Assessment of Disease Activity; PHQ-9, Patient Health Questionnaire – 9 Items; Q1-Fatigue, Single-item Fatigue Question 1; r-axSpA, radiographic axial spondyloarthritis; SF-36, Short Form 36-Item Health Survey; WPAI:SHP, Work Productivity and Activity Impairment Questionnaire- Specific Health Problem.

^a^ Patients only completed the first five questions of the WPAI:SHP questionnaire, which are relevant to these domain scores, if they were employed at the time they entered the study. Therefore, the sample size for these domains was lower. BE MOBILE 1: at Baseline, n=170 for both presenteeism and overall productivities loss scores, and n=188 for absenteeism score; at Week 16, n=158 for both presenteeism and overall productivities loss scores, n=184 for absenteeism score. BE MOBILE 2: at Baseline, n=223 for both presenteeism and overall productivities loss scores, and n=243 for absenteeism score; at Week 16, n=226 for both presenteeism and overall productivities loss scores, and n=241 for absenteeism score.

### **Supplementary Results Table 4. Known-groups validity in pooled studies: Differences in FACIT-Fatigue mean scores between known groups.**

|  |  | **Baseline** | | **Week 16** | |
| --- | --- | --- | --- | --- | --- |
| **Outcome measure** | **Adjacent groups compared** | **Difference in FACIT-Fatigue means between adjacent groups (95% CI)** | **ES** | **Difference in FACIT-Fatigue means between adjacent groups (95% CI)** | **ES** |
| ASQoL Item 7 (“I am tired all the time”) | Yes vs. No | 11.73 (10.27, 13.19) | 1.10 | 14.63 (13.36, 15.90) | 1.37 |
| ASQoL Item 12 (“I get tired easily”) | Yes vs. No | 12.88 (11.15, 14.61) | 1.21 | 12.28 (10.98, 13.59) | 1.15 |
|  |  |  |  |  |  |
| SF-36 Item 9g  (“Did you feel worn out during the past 4 weeks?”) | All vs. Most of the time | 5.85 (3.10, 8.61) | 0.55 | ─ | ─ |
|  | Most^a^ vs. Some of the time | 9.53 (8.05, 11.00) | 0.89 | 11.45 (9.51, 13.40) | 1.07 |
|  | Some vs. A little of the time | 8.25 (6.70, 9.79) | 0.77 | 7.34 (5.94, 8.75) | 0.69 |
|  | A little vs. None of the time | 3.14 (0.78, 5.49) | 0.29 | 4.65 (3.37, 5.94) | 0.44 |
|  |  |  |  |  |  |
| SF-36 Item 9i  (“Did you feel tired during the past 4 weeks?”) | All vs. Most of the time | 4.96 (2.56, 7.35) | 0.46 | 7.84 (4.21, 11.46) | 0.73 |
|  | Most vs. Some of the time | 10.85 (9.44, 12.27) | 1.02 | 9.94 (8.32, 11.57) | 0.93 |
|  | Some vs. A little of the time^b^ | 7.81 (6.17, 9.44) | 0.73 | 7.54 (6.36, 8.73) | 0.71 |
|  | A little vs. None of the time | ─ | ─ | 2.27 (0.40, 4.13) | 0.21 |
|  |  |  |  |  |  |
| PHQ-9 Item 4 (“Over the last 2 weeks, how often have you been bothered by feeling tired or having little energy/”) | Not at all vs. Several days | -2.36 (-4.28, -0.43) | -0.22 | -4.18 (-5.70, -2.65) | -0.39 |
|  | Several days vs. More than half the days | -6.60 (-8.90, -4.29) | -0.62 | -11.95 (-14.49, -9.41) | -1.12 |
|  | More than half the days vs. Nearly every day | -2.10 (-5.82, 1.63) | -0.20 | -2.11 (-7.49, 3.27) | -0.20 |
|  |  |  |  |  |  |
| ASDAS | ID vs. LD | ─ | ─ | -4.69 (-6.65, -2.73) | -0.44 |
|  | LD vs. HD | ─ | ─ | -5.29 (-7.14, -3.44) | -0.50 |
|  | HD^c^ vs. VHD | -6.64 (-8.32, -4.96) | -0.62 | -4.89 (-7.08, -2.71) | -0.46 |

Abbreviations: ASDAS, Ankylosing Spondylitis Disease Activity Score; ASQoL, Ankylosing Spondylitis Quality of Life; CI, confidence interval; ES, effect size; FACIT-Fatigue, Functional Assessment of Chronic Illness Therapy – Fatigue; HD, ASDAS high disease activity; ID, ASDAS inactive disease; LD, ASDAS low disease activity; PHQ-9, Patient Health Questionnaire – 9 Items; SF-36, Short Form 36-Item Health Survey; VHD, ASDAS very high disease activity.

Mean scores of the FACIT-Fatigue were compared between known groups, and the ES of the difference in means between adjacent groups was estimated as the difference in means divided by the overall standard deviation at Baseline.

^a^ At Week 16, the categories ‘All of the time’ and ‘Most of the time’ were collapsed due to a small sample size (n<15) for the category ‘All of the time’.

^b^ At Baseline, the categories ‘A little of the time’ and ‘None of the time’ were collapsed due to a small sample size (n<15) for the category ‘None of the time’.

^c^ At Baseline, the categories ‘ASDAS-LD’ and ‘ASDAS-HD’ were collapsed due to a small sample size (n<15) for the category ‘ASDAS-LD’.

### **Supplementary Results Table 5. Known-groups validity by study: Differences in FACIT-Fatigue mean scores between known groups**

|  |  | **BE MOBILE 1**  **(nr-axSpA)** | | | | **BE MOBILE 2**  **(r-axSpA)** | | | |
| --- | --- | --- | --- | --- | --- | --- | --- | --- | --- |
|  |  | **Baseline** | | **Week 16** | | **Baseline** | | **Week 16** | |
| **Outcome measure** | **Adjacent groups compared** | **Difference in FACIT-Fatigue means between adjacent groups (95% CI)** | **ES** | **Difference in FACIT-Fatigue means between adjacent groups (95% CI)** | **ES** | **Difference in FACIT-Fatigue means between adjacent groups (95% CI)** | **ES** | **Difference in FACIT-Fatigue means between adjacent groups (95% CI)** | **ES** |
| ASQoL Item 7 (“I am tired all the time”) | Yes vs. No | 11.43 (9.11, 13.74) | 1.07 | 14.37 (12.37, 16.38) | 1.35 | 11.92 (10.01, 13.83) | 1.12 | 14.64 (13.00, 16.28) | 1.37 |
| ASQoL Item 12 (“I get tired easily”) | Yes vs. No | 12.86 (10.00, 15.72) | 1.20 | 12.36 (10.23, 14.49) | 1.16 | 12.81 (10.62, 15.01) | 1.20 | 12.01 (10.36, 13.67) | 1.13 |
|  |  |  |  |  |  |  |  |  |  |
| SF-36 Item 9g  (“Did you feel worn out during the past 4 weeks?”) | All vs. Most of the time | 8.66 (4.57, 12.76) | 0.81 | ─ | ─ | 2.66 (-1.13, 6.45) | 0.25 | ─ | ─ |
|  | Most^a^ vs. Some of the time | 8.75 (6.55, 10.94) | 0.82 | 11.35 (8.43, 14.28) | 1.06 | 10.16 (8.16, 12.17) | 0.95 | 11.52 (8.87, 14.16) | 1.08 |
|  | Some vs. A little of the time | 8.85 (6.67, 11.02) | 0.83 | 6.71 (4.50, 8.92 | 0.63 | 7.66 (5.54, 9.78) | 0.72 | 7.78 (5.95, 9.61) | 0.73 |
|  | A little vs. None of the time^b^ | ─ | ─ | 5.35 (3.00, 7.70) | 0.50 | 4.47 (1.87, 7.07) | 0.42 | 4.14 (2.60, 5.67) | 0.39 |
|  |  |  |  |  |  |  |  |  |  |
| SF-36 Item 9i  (“Did you feel tired during the past 4 weeks?”) | All vs. Most of the time | 8.98 (5.59, 12.37) | 0.84 | ─ | ─ | 0.80 (-2.49, 4.10) | 0.08 | ─ | ─ |
|  | Most^c^ vs. Some of the time | 9.14 (6.93, 11.36) | 0.86 | 11.06 (8.62, 13.49) | 1.04 | 12.30 (10.47, 14.14) | 1.15 | 11.20 (9.06, 13.35) | 1.05 |
|  | Some vs. A little of the time^d^ | 7.48 (4.59, 10.38) | 0.70 | 7.68 (5.89, 9.47) | 0.72 | 7.92 (5.93, 9.90) | 0.74 | 7.68 (6.15, 9.21) | 0.72 |
|  | A little vs. None of the time | - | - | - | - | - | - | 1.81 (-0.51, 4.13) | 0.17 |
|  |  |  |  |  |  |  |  |  |  |
| PHQ-9 Item 4 (“Over the last 2 weeks, how often have you been bothered by feeling tired or having little energy/”) | Not at all vs. Several days | -3.97 (-6.94, -1.00) | -0.37 | -3.91 (-6.28, -1.53) | -0.37 | -1.24 (-3.78, 1.29) | -0.12 | -4.31 (-6.31, -2.31) | -0.40 |
|  | Several days vs. More than half the days | -5.79 (-8.80, -2.79) | -0.54 | -12.62 (-15.86, -9.38) | -1.18 | -7.99 (-10.92, -5.05) | -0.75 | -11.76 (-15.22, -8.29) | -1.10 |
|  | More than half the days vs. Nearly every day | - | - | - | - | - | - | - | - |
|  |  |  |  |  |  |  |  |  |  |
| ASDAS | ID vs. LD | - | - | -4.63 (-7.83, -1.44) | -0.43 | - | - | -4.83 (-7.30, -2.36) | -0.45 |
|  | LD vs. HD | - | - | -5.11 (-8.02, -2.19) | -0.48 | - | - | -5.42 (-7.82, -3.02) | -0.51 |
|  | HD^e^ vs. VHD | -6.48 (-9.04, -3.92) | -0.61 | -6.11 (-9.34, -2.88) | -0.57 | -6.80 (-9.03, -4.56) | -0.64 | -3.37 (-6.36, -0.38) | -0.32 |

Abbreviations: ASDAS, Ankylosing Spondylitis Disease Activity Score; HD, ASDAS high disease activity; ID, ASDAS inactive disease; LD, ASDAS low disease activity; VHD, ASDAS very high disease activity; ASQoL, Ankylosing Spondylitis Quality of Life; ES, effect size; FACIT-Fatigue, Functional Assessment of Chronic Illness Therapy – Fatigue; nr-axSpA, non-radiographic axial spondyloarthritis; PHQ-9, Patient Health Questionnaire – 9 Items; r-axSpA, radiographic axial spondyloarthritis; SF-36, Short Form 36-Item Health Survey.

Mean scores of the FACIT-Fatigue were compared per known group, and the ES of the difference in means between adjacent groups was estimated as the difference in means divided by the overall standard deviation at Baseline.

^a^ For both trials, at Week 16, the categories ‘All of the time’ and ‘Most of the time’ were collapsed due to a small sample size (n<15) for the category ‘All of the time’.

^b^ For BE MOBILE 1, at Baseline, the categories ‘A little of the time’ and ‘None of the time’ were collapsed due to a small sample size (n<15) for the category ‘None of the time’.

^c^ For both trials, at Week 16, the categories ‘All of the time’ and ‘Most of the time’ were collapsed due to a small sample size (n<15) for the category ‘All of the time’.

^d^ For both trials, at Baseline and/or Week 16, the categories ‘A little of the time’ and ‘None of the time’ were collapsed due to a small sample size (n<15) for the category ‘None of the time’.

^e^ For both trials, at Baseline, the categories ‘ASDAS-LD’ and ‘ASDAS-HD’ were collapsed due to a small sample size (n<15) for the category ‘ASDAS-LD’.

### **Supplementary Results Table 6. Responsiveness in pooled studies: Changes in FACIT-Fatigue score by criterion measure change category.**

|  | **Improvement** | | | **No** **Improvement** | |
| --- | --- | --- | --- | --- | --- |
| **Criterion measure and**  **Statistic** | **Improvement by 3 Levels** | **Improvement by 2 Levels** | **Improvement by 1 Level** | **No Change** | **Worsening by ≥1 Level** |
| **SF-36 Item 9i ^a^** | | | | | |
| n | 0 | 70 | 197 | 233 | 67 |
| Least Square Mean Change (95% CI) ^b^ | - | 16.17 (14.52, 17.82) | 9.07 (8.10, 10.04) | 4.26 (3.37, 5.16) | 0.02 (-1.64, 1.68) |
| ES ^c^ | - | 1.51 | 0.85 | 0.40 | 0.00 |
| **ASAS Response Levels ^d^** | | | | | |
| n | 96 | 118 | 113 | 231 | |
| Least Square Mean Change (95% CI) ^b^ | 13.38 (11.97, 14.80) | 11.18 (9.90, 12.45) | 6.15 (4.85, 7.45) | 2.61 (1.70, 3.52) | |
| ES ^c^ | 1.25 | 1.05 | 0.58 | 0.24 | |
| **ASDAS Disease States ^e^** | | | | | |
| n | 35 | 89 | 209 | 227 | |
| Least Square Mean Change (95% CI) ^b^ | 14.88 (12.46, 17.31) | 12.32 (10.79, 13.84) | 7.13 (6.14, 8.12) | 3.49 (2.53, 4.44) | |
| ES ^c^ | 1.39 | 1.15 | 0.67 | 0.33 | |

Abbreviations: ASAS, Assessment of SpondyloArthritis international Society; ASDAS, Ankylosing Spondylitis Disease Activity Score; CI, confidence interval; ES, effect size; FACIT-Fatigue, Functional Assessment of Chronic Illness Therapy - Fatigue; SF-36, Short Form 36-Item Health Survey.

^a^ SF-36 Item 9i asks patients to answer, “How much of the time during the past 4 weeks did you feel tired?” by selecting from the following: “All the time”, “Most of the time”, “Some of the time”, “A little of the time”, “None of the time”.

^b^ Least squares mean change scores (95% CI) and p-values were estimated from the analysis of covariance model, including change from Baseline in the target total score at Week 16 as the dependent variable and changes in a given anchor as the independent variable, adjusting for Baseline score.

^c^ ES estimated as the least squares mean change divided by overall standard deviation at Baseline.

^d^ Patients who met ASAS-PR response criteria but did not meet ASAS20 and ASAS40 response criteria as well as patients who met both ASAS20 and ASAS-PR response criteria but did not meet ASAS40 response criteria were excluded from the analysis. Patients were categorized into mutually exclusive groups corresponding to: 1) patients who did not meet any of the three ASAS response criteria (ASAS20 non-responder; ASAS40 non-responder; ASAS-PR non-responder); 2) patients who met ASAS20 response criteria, but did not meet ASAS40 and ASAS-PR response criteria (ASAS20 responder; ASAS40 non-responder; ASAS-PR non-responder); 3) patients who met ASAS40 response criteria but did not meet ASAS-PR response criteria (ASAS40 responder; ASAS-PR non-responder); 4) patients who met both ASAS40 and ASAS-PR response criteria (ASAS40 responder; ASAS-PR responder).

^e^ Patients were categorized into mutually exclusive groups corresponding to their change in ASDAS disease states. ASDAS score <1.3 indicated inactive disease, ≥1.3 to <2.1 indicated low disease activity, ≥2.1 to ≤3.5 indicated high disease activity, and >3.5 indicated very high disease activity.

### **Supplementary Results Table 7A. Responsiveness by study: Spearman’s correlations between changes in FACIT-Fatigue score and changes in selected criterion measures.**

|  | **BE MOBILE 1**  **(nr-axSpA)** | | **BE MOBILE 2**  **(r-axSpA)** | |
| --- | --- | --- | --- | --- |
| **Criterion measure** | **n** | **r** | **n** | **r** |
| ASAS Response Levels | 241 | -0.41 | 317 | -0.40 |
| ASDAS Disease States | 244 | -0.36 | 316 | -0.36 |
| ASDAS-CII/ASDAS-MI | 244 | -0.40 | 316 | -0.40 |
| SF-36 Item 9g | 246 | -0.59 | 321 | -0.53 |
| SF-36 Item 9i | 246 | -0.58 | 321 | -0.52 |
| PHQ-9 Item 4 | 246 | -0.35 | 321 | -0.21 |

All correlations were statistically significant at p<0.001.

Abbreviations: ASAS, Assessment of SpondyloArthritis international Society; ASDAS, Ankylosing Spondylitis Disease Activity Score; CI, confidence interval; FACIT-Fatigue, Functional Assessment of Chronic Illness Therapy - Fatigue; PHQ-9, Patient Health Questionnaire – 9 Items; nr-axSpA, non-radiographic axial spondyloarthritis; r-axSpA, radiographic axial spondyloarthritis; SF-36, Short Form 36-Item Health Survey.

### **Supplementary Results Table 7B. Responsiveness by study: Changes from Baseline to Week 16 in FACIT-Fatigue score by retained criterion measure level of change.**

|  |  | **Improvement** | | | | | **No** **Improvement** | |
| --- | --- | --- | --- | --- | --- | --- | --- | --- |
| **Study** | **Criterion measure and**  **Statistic** | **Improvement by 3 Levels** | **Improvement by 2 Levels** | | **Improvement by 1 Level** | | **No Change** | **Worsening by ≥1 Level** |
| **BE MOBILE 1**  **(nr-axSpA)** | **SF-36 Item 9i ^a^** | | | | | | | |
|  | n | - | | 31 | | 83 | 98 | 34 |
|  | Least Square Mean Change (95% CI) ^b^ | - | | 16.95 (14.48, 19.43) | | 8.77 (7.26, 10.27) | 3.45  (2.07, 4.84) | -0.19  (-2.55, 2.16) |
|  | ES ^c^ | - | | 1.59 | | 0.82 | 0.32 | -0.02 |
|  | **ASAS Response Levels ^d^** | | | | | | | |
|  | n | 37 | | 51 | | 44 | 109 | |
|  | Least Square Mean Change (95% CI) ^b^ | 13.79 (11.46, 16.13) | | 11.50 (9.53, 13.47) | | 6.26 (4.14, 8.38) | 1.92 (0.56, 3.27) | |
|  | ES ^c^ | 1.29 | | 1.08 | | 0.59 | 0.18 | |
|  | **ASDAS Disease States ^e^** | | | | | | | |
|  | n | 17 | | 37 | | 78 | 112 | |
|  | Least Square Mean Change (95% CI) ^b^ | 13.68 (10.05, 17.30) | | 12.60 (10.12, 15.07) | | 6.93 (5.23, 8.62) | 3.04 (1.62, 4.45) | |
|  | ES ^c^ | 1.28 | | 1.18 | | 0.65 | 0.28 | |
|  |  |  | |  | |  |  | |
| **BE MOBILE 2**  **(r-axSpA)** | **SF-36 Item 9i ^a^** | | | | | | | |
|  | n | - | | 39 | | 114 | 135 | 33 |
|  | Least Square Mean Change (95% CI) ^b^ | - | | 15.42 (13.21, 17.63) | | 9.27 (8.00, 10.54) | 4.89 (3.72, 6.07) | 0.29 (-2.06, 2.64) |
|  | ES ^c^ | - | | 1.45 | | 0.87 | 0.46 | 0.03 |
|  | **ASAS Response Levels ^d^** | | | | | | | |
|  | n | 59 | | 67 | | 69 | 122 | |
|  | Least Square Mean Change (95% CI) ^b^ | 13.10 (11.32, 14.87) | | 10.93 (9.25, 12.60) | | 6.02 (4.38, 7.66) | 3.29 (2.05, 4.52) | |
|  | ES ^c^ | 1.23 | | 1.02 | | 0.56 | 0.31 | |
|  | **ASDAS Disease States ^e^** | | | | | | | |
|  | n | 18 | | 52 | | 131 | 115 | |
|  | Least Square Mean Change (95% CI) ^b^ | 16.08 (12.80, 19.36) | | 12.10 (10.17, 14.02) | | 7.18 (5.97, 8.40) | 4.01 (2.71, 5.30) | |
|  | ES ^c^ | 1.51 | | 1.13 | | 0.67 | 0.38 | |

Abbreviations: ASAS, Assessment of SpondyloArthritis international Society; ASDAS, Ankylosing Spondylitis Disease Activity Score; CI, confidence interval; ES, effect size; FACIT-Fatigue, Functional Assessment of Chronic Illness Therapy - Fatigue; nr-axSpA, non-radiographic axial spondyloarthritis; r-axSpA, radiographic axial spondyloarthritis; SF-36, Short Form 36-Item Health Survey.

^a^ SF-36 Item 9i asks patients to answer, “How much of the time during the past 4 weeks did you feel tired?” by selecting from the following: “All the time”, “Most of the time”, “Some of the time”, “A little of the time”, “None of the time”.

^b^ Least squares mean change scores (95% CI) and p-values were estimated from the analysis of covariance model, including change from Baseline in the target total score at Week 16 as the dependent variable and changes in a given criterion measure as the independent variable, adjusting for Baseline score.

^c^ ES estimated as the least squares mean change divided by overall standard deviation at Baseline.

^d^ Patients who met ASAS-PR response criteria but did not meet ASAS20 and ASAS40 response criteria as well as patients who met both ASAS20 and ASAS-PR response criteria but did not meet ASAS40 response criteria were excluded from the analysis. Patients were categorized into mutually exclusive groups corresponding to: 1) patients who did not meet any of the three ASAS response criteria (ASAS20 non-responder; ASAS40 non-responder; ASAS-PR non-responder); 2) patients who met ASAS20 response criteria, but did not meet ASAS40 and ASAS-PR response criteria (ASAS20 responder; ASAS40 non-responder; ASAS-PR non-responder); 3) patients who met ASAS40 response criteria but did not meet ASAS-PR response criteria (ASAS40 responder; ASAS-PR non-responder); 4) patients who met both ASAS40 and ASAS-PR response criteria (ASAS40 responder; ASAS-PR responder).

^e^ Patients were categorized into mutually exclusive groups corresponding to their change in ASDAS disease states. ASDAS score <1.3 indicated inactive disease, ≥1.3 to <2.1 indicated low disease activity, ≥2.1 to ≤3.5 indicated high disease activity, and >3.5 indicated very high disease activity.

### **Supplementary Results Table 8. Meaningful within-patient change thresholds for pooled studies: Baseline to Week 16 changes in FACIT-Fatigue score.**

|  | Improvement | | | No Improvement | |
| --- | --- | --- | --- | --- | --- |
| **Criterion measure and Statistic** | **Improvement by 3 Levels** | **Improvement by 2 Levels** | **Improvement by 1 Level** | **No Change** | **Worsening (collapsed categories)** |
| **SF-36 Item 9i ^a^** | **CFB = -3** | **CFB = -2** | **CFB = -1** | **CFB = 0** | **CFB ≥1** |
| n | 0 | 70 | 197 | 233 | 67 |
| Mean (95% CI) | - | 18.07 (15.64, 20.50) | 9.57 (8.48, 10.66) | 3.49 (2.55, 4.44) | -0.76 (-2.42, 0.90) |
| Median | - | 19.00 | 9.00 | 3.00 | 0.00 |
| Min, Max | - | -1.00, 48.00 | -16.00, 41.00 | -21.00, 24.00 | -19.00, 17.00 |
| ES | - | 1.69 | 0.90 | 0.33 | -0.07 |
| **ASAS Response Levels ^b^** | **ASAS40 and ASAS-PR responders** | **ASAS40 responder**  **ASAS-PR non-responder** | **ASAS20 responder**  **ASAS40 non-responder**  **ASAS-PR non-responder** | **ASAS20 non-responder**  **ASAS40 non-responder**  **ASAS-PR non-responder** | |
| n | 96 | 118 | 113 | 231 | |
| Mean (95% CI) | 12.27 (10.19, 14.36) | 11.76 (10.10, 13.43) | 5.88 (4.65, 7.11) | 2.91 (1.81, 4.01) | |
| Median | 10.50 | 11.00 | 5.00 | 2.00 | |
| Min, Max | -4.00, 48.00 | -7.00, 35.00 | -7.00, 27.00 | -21.00, 25.00 | |
| ES | 1.15 | 1.10 | 0.55 | 0.27 | |
| **ASDAS Disease States ^c^** | **3 levels of improvement in disease state** | **2 levels of improvement in disease state** | **1 level of improvement in disease state** | **No change or ≥1 level of worsening in disease state** | |
| n | 35 | 89 | 209 | 227 | |
| Mean (95% CI) | 15.17 (11.89, 18.46) | 11.54 (9.31, 13.77) | 7.24 (6.05, 8.44) | 3.64 (2.60, 4.69) | |
| Median | 13.00 | 10.00 | 6.00 | 3.00 | |
| Min, Max | 1.00, 40.00 | -7.00, 48.00 | -19.00, 34.00 | -18.00, 31.00 | |
| ES | 1.42 | 1.08 | 0.68 | 0.34 | |

Abbreviations: ASAS, Assessment of Spondyloarthritis international Society; ASAS-PR, ASAS-partial remission; ASDAS, Ankylosing Spondylitis Disease Activity Score; CFB, change from Baseline; CI, confidence interval; FACIT-Fatigue, Functional Assessment of Chronic Illness Therapy - Fatigue; Max, maximum; Min, minimum; SF-36, Short Form 36-Item Health Survey.

All effects were statistically significant at p<0.001.

^a^ SF-36 Item 9i asks patients to answer, “How much of the time during the past 4 weeks did you feel tired?” by selecting from the following: “All the time”, “Most of the time”, “Some of the time”, “A little of the time”, “None of the time”

^b^ Patients were categorized into mutually exclusive groups corresponding to: 1) patients who did not meet any of the three ASAS response criteria (ASAS20 non-responder; ASAS40 non-responder; ASAS-PR non-responder); 2) patients who met ASAS20 response criteria, but did not meet ASAS40 and ASAS-PR response criteria (ASAS20 responder; ASAS40 non-responder; ASAS-PR non-responder); 3) patients who met ASAS40 response criteria but did not meet ASAS-PR response criteria (ASAS40 responder; ASAS-PR non-responder); 4) patients who met both ASAS40 and ASAS-PR response criteria (ASAS40 responder; ASAS-PR responder). ASAS20 is defined as improvement of ≥20% and an absolute improvement of ≥1 unit on a 0–10 numeric rating scale in ≥3 of the four ASAS domains. In addition, ASAS20 requires no worsening in the remaining domain (relative worsening of ≥20% and absolute worsening of ≥1 unit). ASAS40 is defined as a relative improvement of ≥40% and an absolute improvement of ≥2 units on a 0–10 NRS in ≥3 of the four ASAS domains and no worsening at all in the remaining domain. ASAS-PR is defined as a score of ≤2 units on a 0–10 NRS in all four domains.

^c^ Patients were categorized into mutually exclusive groups corresponding to their change in ASDAS disease states. ASDAS score <1.3 indicated inactive disease, ≥1.3 to <2.1 indicated low disease activity, ≥2.1 to ≤3.5 indicated high disease activity, and >3.5 indicated very high disease activity.

### **Supplementary Results Table 9. Fatigue severity bands for the pooled studies: Logistic regression statistics.**

| Item (Criterion measure) | Number of Responders ^a^ | Number of Observations ^b^ | Cutoff Corresponding to Maximum YI | Maximum YI | Sensitivity, Specificity | Percent Correctly Classified | AUC (95% CI) |
| --- | --- | --- | --- | --- | --- | --- | --- |
| **SF-36 Item 9g** |  |  |  |  |  |  |  |
| 1 vs 2-4 ^c^ | 460 | 1153 | 37.00 | 0.62 | 0.85, 0.78 | 80.5% | 0.89 (0.87, 0.91) |
| 1-2 vs 3-4 ^c^ | 856 | 1153 | 31.00 | 0.66 | 0.81, 0.86 | 82.1% | 0.92 (0.90, 0.93) |
| 1-3 vs 4 ^c^ | 1114 | 1153 | 28.00 | 0.66 | 0.74, 0.92 | 74.3% | 0.91 (0.87, 0.95) |
| **SF-36 Item 9i** |  |  |  |  |  |  |  |
| 1 vs 2-4 ^c^ | 309 | 1153 | 41.00 | 0.66 | 0.83, 0.83 | 82.8% | 0.91 (0.89, 0.93) |
| 1-2 vs 3-4 ^c^ | 725 | 1153 | 33.00 | 0.65 | 0.83, 0.82 | 82.5% | 0.91 (0.89, 0.92) |
| 1-3 vs 4 ^c^ | 1077 | 1153 | 26.00 | 0.57 | 0.80, 0.78 | 79.7% | 0.87 (0.84, 0.91) |
| **ASDAS Disease States** |  |  |  |  |  |  |  |
| 1 vs 2-4 ^d^ | 103 | 1721 | 42.00 | 0.57 | 0.86, 0.71 | 71.5% | 0.85 (0.81, 0.88) |
| 1-2 vs 3-4 ^d^ | 354 | 1721 | 38.00 | 0.41 | 0.77, 0.64 | 66.9% | 0.77 (0.75, 0.80) |
| 1-3 vs 4 ^d^ | 1129 | 1721 | 31.00 | 0.33 | 0.77, 0.56 | 69.5% | 0.73 (0.70, 0.75) |

Abbreviations: ASDAS, Ankylosing Spondylitis Disease Activity Score; ASDAS-HD, ASDAS-high disease activity; ASDAS-ID, ASDAS-inactive disease; ASDAS-LD, ASDAS-low disease activity; ASDAS-VHD, ASDAS-very high disease activity; AUC, area under the curve; CI, confidence interval; FACIT-Fatigue, Functional Assessment of Chronic Illness Therapy - Fatigue; SF-36, Short Form 36-Item Health Survey; YI, Youden index.

^a^ Number of observations across all time points included in each analysis meeting responder criteria.

^b^ Number of observations across the time points included in each analysis.

^c^ 1 = None of the time / A little of the time; 2 = Some of the time; 3 = Most of the time; 4 = All of the time.

^d^ 1 = ASDAS-ID; 2 = ASDAS-LD; 3 = ASDAS-HD; 4 = ASDAS-VHD.
